# Spatial Analyses of Mono, Di and Trinucleotide Trends in Plant Genes

**DOI:** 10.1371/journal.pone.0022855

**Published:** 2011-08-01

**Authors:** Andrea Porceddu, Salvatore Camiolo

**Affiliations:** Dipartimento di Scienze Agronomiche e Genetica Vegetale Agraria, Università degli Studi di Sassari, Sassari, Italy; University of Umeå, Sweden

## Abstract

Genomic DNA sequences display compositional heterogeneity on many scales. In this paper we analyzed tendencies and anomalies in the occurence of mono, di and trinucleotides in structural regions of plant genes. Representation of these trends as a function of position along genic sequences highlighted compositional features peculiar of either monocots or eudicots that were remarkably uniform within these two evolutionary clades. The most evident of these features appeared in the form of gradient of base content along the direction of transcription. The robustness of such a representation was validated in sequences sub-datasets generated considering structural and compositional features such as total length of cds, overall GC content and genic orientation in the genome. Piecewise regression analyses indicated that the gradients could be conveniently approximated to a two segmented model where a first region featuring a steep slope is followed by a second segment fitting a milder variation. In general, monocots species showed steeper segments than eudicots. The guanine gradient was the most distinctive feature between the two evolutionary clades, being moderately increasing in eudicots and firmly decreasing in monocots. Single gene investigation revealed that a high proportion of genes show compositional trends compatible with a segmented model suggesting that these features are essential attributes of gene organization. Dinucleotide and trinucleotide biases were referred to expectation based on a random union of the component elements. The average bias at dinucleotide level identified a significant undererpresentation of some dinucleotide and the overrepresention of others. The bias at trinucleotide level was on average low. Finally, the analysis of bryophyte coding sequences showed mononucleotide, dinucleotide and trinucleotide compositional trends resembling those of higher plants. This finding suggested that the emergenge of compositional bias is an ancient event in evolution which was already present at the time of land conquest by green plants.

## Introduction

Compositional heterogeneity is a common feature of eukaryotic genomes [1]–[3]. Many biochemical and molecular studies have focused on descriptions of the structural organization of such a variation at different genomic scales. Large blocks of DNA of homogeneous G+C content were described in warm blooded vertebrates as the main high-scale compositional units [4]. These blocks, termed isochores, proved to be strongly associated with the genome organization [5]. Indeed observations from the mammalian genomes indicated that isochores correlated with gene density [4], repetitive DNA elements distribution [6], chromosomal bands [7], [8] and potentially also with replication time in the genome [7]. Computational studies in the human genome have illustrated that the frequencies of di and tri nucleotides provide a characteristic pattern of different isochore families and such a short sequence design is credited to be relevant for various structural and functional aspects of genome biology [9]. Studies conducted on long genomic sequences have extended the perception of compositional heterogeneity to many multicellular organisms belonging to a wide variety of evolutionary taxa, indicating, however, the existence of large genomic regions which do not fit with the classic isochore model [10].

The availability of many genomic sequences has provided us with the unprecedented opportunity to perform compositional studies at low-scale level. These analyses are, in time, highlighting a number of genomic features associated to basic cellular mechanisms. Huvet and co-workers [11] have recently reported that in more than one quarter of the human genome the nucleotide compositional skew presents characteristic patterns consisting of succession of “N-shaped” profiles. Based on these observations the authors have proposed a new model of gene organization which integrates transcription, replication, and chromatin structure [11].

Other features, that have not yet found a clear causal link, are currently interpreted as punctuation marks for low-scale genomic organization. For example, spikes in GC content have been associated to the boundaries of transcriptional units in warm blooded and invertebrate species [12]. An analogous feature seems to be present in plants and fungi genomes where spikes of GC compositional strand bias identify transcriptional start sites [13].

Analyses at intragenic scale level have mainly focused on non random usage of synonymous codons. Reports from several species have revealed a relation between synonymous codon usage and position in the coding sequence. For example, enterobacterial genes avoid some codons near the start sites, perhaps, to limit the formation of secondary structures in the messenger which could interfere with ribosome binding site near the start of translation [14].

Qin and co-workers have demonstrated how the pattern of codon usage bias along genes may have different features among species [15]. In yeast and several prokaryotic species it increases along translational direction which is consistent with purifying selection against nonsense errors. *Drosophila melanogaster* codon usage bias is high at the ends and lower in the middle of coding sequence probably as a consequence of the Hill Robertson effect.

Several analyses conducted on coding and genic sequence have indicated the existence of a base compositional bias at the termini of plant genes [16], [17]. Various explanations have been proposed for such bias, including bias in codon and aminoacid usage, and mutation related process. However, peculiarities of each species and intrinsic limitations of the experimental setups have, so far, prevented the convergence of this data on a well defined picture. Niimura and co-workers showed that base appearance at the codon third position of the terminal regions of both *Arabidopsis* and *Oryza* genes is extremely biased [16]. Unfortunately as the analysis considered exclusively the third codon position, mainly the involvement of bias in synonymous codon usage could be tested. Wong and co-workers have reported that in the first 1.5 kb of monocots but not eudicots coding sequences there is a negative gradient of G+C content proceeding along the translation direction [17]. This compositional bias was observed, although with different intensity, for all three codon bases and therefore affected both synonymous codon and aminoacid usages. In this work we present a detailed analysis of compositional bias as function of position in structural regions of plant genes. The investigation considered three different degree of compositional complexity, from mono-nucleotide to trinucleotides and for each of these, the biases were calculated under the hypothesis of random union of the component units. The emerging picture offers novel elements which will be instrumental for defining the compositional signatures of plant genes.

## Materials and Methods

### Sequence datasets

DNA sequences of two eudicots (*Arabidopsis thaliana*, *Vitis vinifera*) and two monocots (*Oryza sativa* and *Brachypodium distaychon*.) were used in this study. All datasets were filtered out for i) transposons and ii) pseudogenes sequences, iii) mitochondrial and chloroplast genes. The cDNA sequences of *Vitis vinifera*, were downloaded from the NCBI FTP server (ftp://ftp.ncbi.nih.gov/). The genomic sequence of *Arabidopsis thaliana* (TAIR 8) and rice (v6.1) were downloaded from the http://www.arabidopsis.org and ftp://ftp.plantbiology.msu.edu/pub/data/Eukaryotic_Projects/o_sativa/annotation_dbs/pseudomolecules/version_6.1/ respectively. The cDNA sequences of *Brachypodium* were retrieved from http://ftp.brachypodium.org/files/Annotation/ [18]. The number of transcripts used for the analysis were 23536 for *Arabidopsis thaliana*, 31088 for *Oryza*, 32255 for *Brachypodium* and 56478 for *Vitis*. *Physcomitrella* (30866) and *Selaginella* (27560) coding sequences were retrieved from the EMBL-Coding sequences database [19].

### Ensemble graphs

#### Mononucleotide

The compositional profiles along sequences were computed using either sliding or adjacent windows of different sizes (33, 51 and 99 bases).

The resulting pictures were substantially unchanged independently from the size of the window and the step between two consecutive windows. The ensemble profiles were generated by averaging the corresponding base content of each window of all genes at each position along the sequences. For the analysis carried out on genic sequences and referred to genomic coordinates only the counts of windows without masked nucleotides were considered. The analyses were carried out both along and opposite to the translational direction using the start and stop codons as reference, respectively.

#### Dinucleotide

Dinucleotide bias was estimated through the odds ratio [3] ρ = f(XY)/f(X)*f(Y) where f(X) and f(Y) denotes the frequencies of the nucleotide X and Y at respectively, and f(XY) is the frequency of the dinucleotide XY in the sequence window under study.

Dinucleotides frequencies at different frames were computed considering the base codon indicated by the frame subscript. For example for dinucleotides 1_2 it was considered the first and second base of each codon. Accordingly the ρ values were calculated taking into account base frequency at the positions indicated by the frame subscript.

#### Trinucleotide

Trinucleotide bias γXYZ [3] was estimated through the odds ratio f(XYZ)*(f(X)*f(Y)*f(Z))/f(XY)*f(YZ)*f(XNZ)), where f(XYZ) is the frequency of the trinucleotide XYZ, f(X), f(Y) and f(Z) are the frequencies of mononucleotides, and f(XY), f(YZ), f(XNZ) are the frequencies of the dinucleotides identifying the given trinucleotide.

For trinucleotides of different frames (1_2_3, 2_3_1 and 3_1_2) we used the same procedures explained for dinucleotides.

### Segmented regression

Segmented or piecewise regression is the process of fitting data to possible more than one linear function. To calculate segmented regression we wrote a C program that selects the most statistically significant linear model that consists of up to two linear equations and calculates the values of the independent variable where the slopes of the linear functions change (breakpoint). The models identified were adopted only when the improvement in explanation of data over simple regression could not have arisen by chance. To test this hypothesis we used the ANOVA procedure according to Ryan et al [20].

## Results

To gather a first insight on spatial compositional patterns of plant genes we studied the global profile of mononucleotides as a function of position along genic sequences of two monocots (*Oryza sativa* and *Brachypodium distachyon*) and two eudicots species (*Arabidopsis thaliana*, *Vitis vinifera*).

### Coding sequence trends

The mononucleotide graphs revealed interesting compositional features that in some cases allowed to distinguish between monocots and dicots species.

#### Eudicots

**Figure 1:**
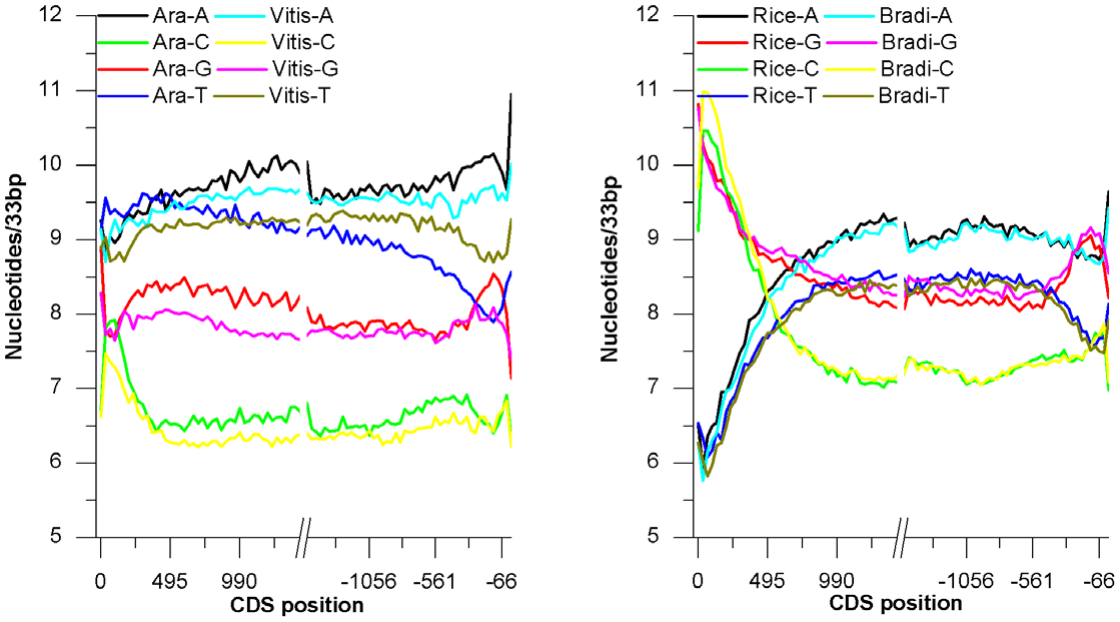
Mononucleotide content of CDS. Overall mononucleotides content as function of position in eudocots (*Vitis* and *Arabidopsis*) and monocots (*Oryza* and *Brachypodium*) coding sequences and averaged over all sequences in the dataset with a 33 bp adiacent window.

Guanine content of eudicots genes increased along the direction of translation to reach soon a plateau and then slowly decreased for the whole length of the sequence with the exception of the 3' end where a positive spike was evident (figure 1a). Cytosine profiles steeply decreased along the direction of translation to reach a plateau level which was modified only in the second half of the gene leaving place to a steadily increasing trend (figure 1a). Adenine profiles were the most regular of eudicots genes increasing steadily throughout the whole gene length. Finally thymine profiles, first increased and then steadily decreased along the direction of translation (figure 1a).

#### Monocots

Guanine and cytosine depicted concave-shaped profiles in all analyzed monocots species (figure 1b). The higher content of guanine over cytosine was almost uniformly distributed over the whole length of monocots coding sequences with the exception of the 5' end region where cytosines were more frequent than guanines.

Nearly convexes profiles were observed for both adenine and thymine, with the adenine content being almost constantly higher than thymine. The only exception to such a behaviour was again at the 5' end of the cds where the two profiles were nearly identical.

**Figure 2:**
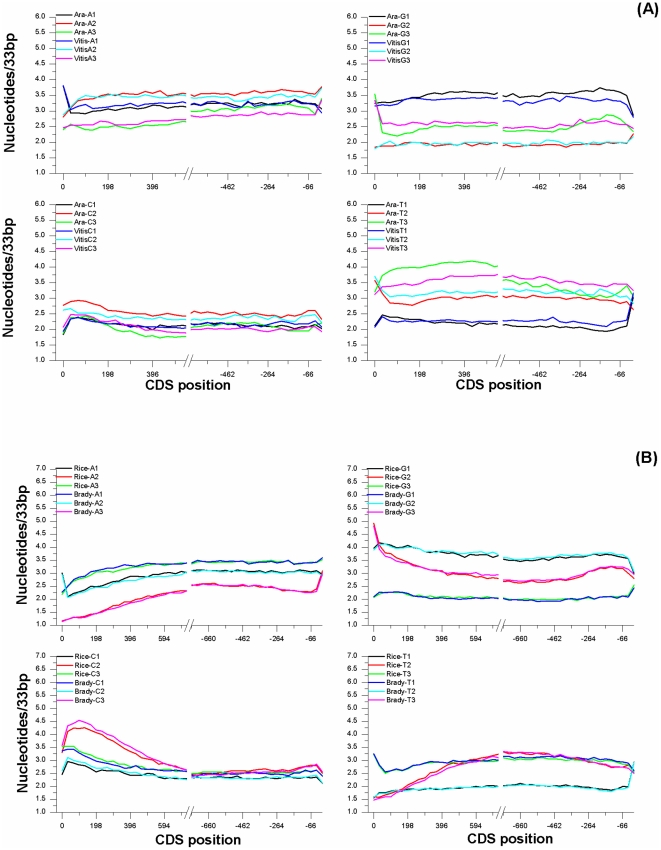
Contribution of the three nucleotide codon positions. Overall first, second and third codon nucleotide content as function of position in eudicots(a) and monocots(b) coding sequences and averaged over all sequences in the dataset with a 33 bp adiacent window.

To analyze the contribution of different codon bases to the compositional profiles of figure 1, each point of the plots was resolved in three components attributable to the first second and third base of codons (figure 2a–b).

For each base, the graphs for the first, second and third position of the codon depicted a similar shape. In all cases, most of total base variation was attributable to the third codon positions followed by the second and first codon positions. Such an effect was particularly evident for thymine followed by cytosine and adenine and at last guanine.

### Ensemble graphs are representative of single gene trends

Because the above graphs were constructed by considering the ensemble averages of single window positions it is important to verify that they describe the general trends of genes and do not represent artefacts due to compositional differences between groups of genes distinguishable for structural or genomic features. The whole analysis was therefore repeated for the most deeply annotated *Arabidopsis* and *Oryza* on sequences datasets partitioned based on various criteria (gene length, orientation on helix and GC content of coding sequences). The relations between base composition and absolute position in coding sequence (i.e. the distance of each window from the first translation codon) were similar across datasets partitioned in length classes (figures S1, S2). These findings ruled out the hypothesis that the ensemble graphs could have reproduced artefacts due to compositional differences between genes of different length.

Other partition criteria considered the orientation of genes in the chromosomes (i.e forward or reverse) and total G+C content of coding sequences. In all cases the shape of the trends was qualitatively similar to those observed for the original dataset (see Supplemental figures S3, S4, S5, S6).

The analysis was then scaled at single gene level. Because most of the ensemble trends could be fitted by two-linear models, we analyzed single gene trends by segmented regression. This analysis returns the segmented linear model which allows the largest improvement in explanation of data over the single linear regression. As a matter of fact, an high proportion (between 61.9 and 70.7%) of single gene trends were better described in the first two kb of length by complex functions with two linear rather than by a simple linear regression (Tables 1, 2 and S1). A combined analysis of the slopes of the fitted segments and of the position of breakpoints indicated that a high proportion of single gene models were compatible with the ensemble graphs (see also Figure S7). Based on these results we conclude that the ensemble models are *bona fide* representations of genic trends.

**Table 1:** Break points and slopes of the ensemble graphs for *Arabidopsis thaliana*.

	Break Point	Slope 1	Slope 2
**A**	–	0.68	–
**G**	14.4	0.86	−0.29
**C**	11.7	−2.96	0.00
**T**	10.4	0.88	−0.43

Slopes are expressed in bp/Kbp.

**Table 2:** Percentages of the Arabidopsis coding sequences that significantly fitted the segmented regression and the linear models.

					Slope 1						
	Positive					Negative					
	A	G	C	T		A	G	C	T		
	4.6	2.9	2.7	1.1		13.0	18.0	33.9	14.3	**Positive**	
**Segmented Regression**											**Slope 2**
	21.2	21.5	13.6	22.0		0.8	2.9	3.8	3.9	**Negative**	
**Linear Regression**	12.9	4.2	5.7	2.6		1.3	5.3	4.4	10.4		
**Not Fitted**	46.2	45.1	35.9	45.6							

Icons represent either positive or negative trends with respect to the breakpoint.

### Trends of genic untranslated sequences

#### UTR

The 5' UTR graphs were aligned taking as reference the nucleotide preceding the translation starting codon. The gradients were, in general, very mild with the exception of the last windows which showed the most significant variations (see Figure S8) indicating that there are striking differences in base composition at the boundary between the 5'UTR and the coding sequences.

3'UTR trends, calculated for 500 bp sequences after the translation stop codon, were barely detectable. The most relevant variations were observed for the thymine content in *Arabidopsis* which decreased proceeding toward the 3'end (Figure S8).

#### Introns

**Figure 3:**
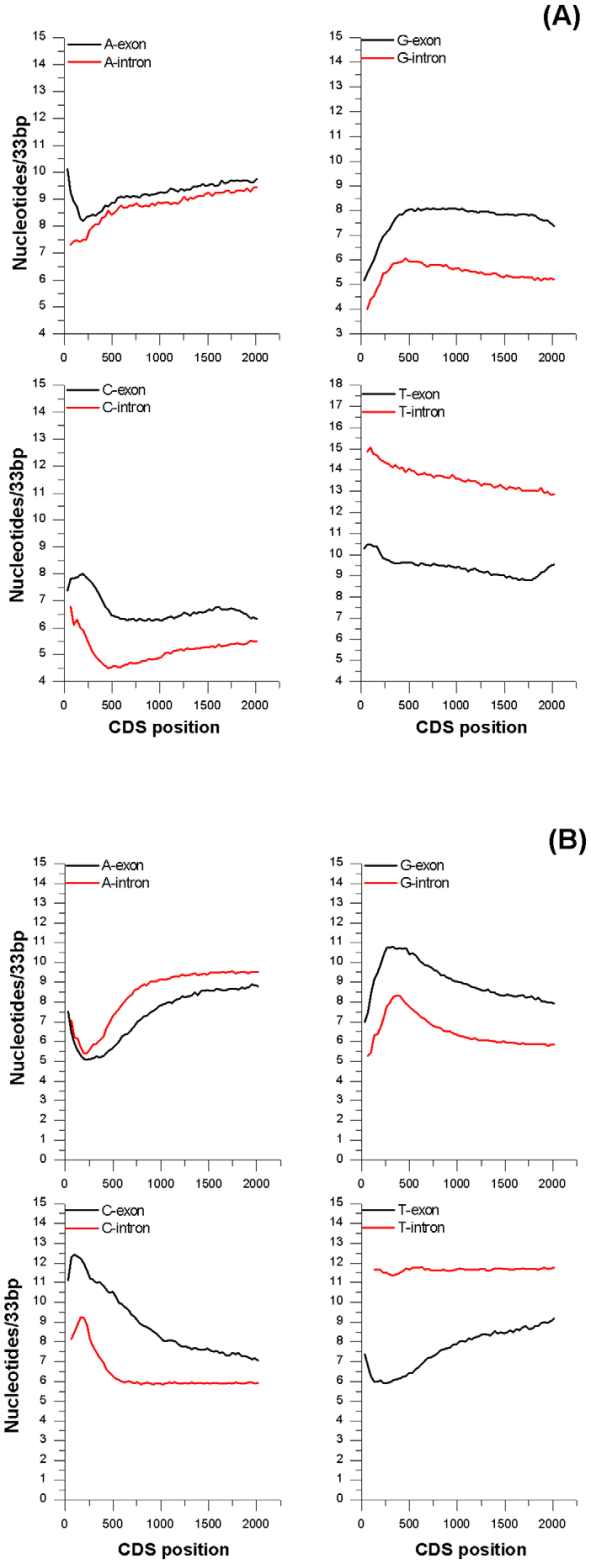
Exon and intron compositional gradients referred to genomic coordinate. Mononucleotide content of intron and exon. The adjacent windows were of 33 bp.

To compare the compositional gradients of introns to those of CDS, ensemble averages were calculated on genic sequences with exon masked. The graph for both *Arabidopsis* and rice showed trends resembling those observed for coding sequences. It is worth remembering that this experimental setup evaluates intron's composition as function of genomic position whereas CDS ensembles are referred to cDNA coordinates. To study exon graphs as a function of position in genes we analyzed genomic sequences after intron masking. The ensemble graphs referred to genomic coordinates are reported in figure 3a–b. Beside the expected differences in the intercepts, due to compositional diversity between introns and exons, the two trends were remarkably similar.

### Dinucleotide trends

As next step in our analyses we studied dinucleotide bias as function of position in genic sequences. The average dinucleotide bias at each position was analyzed using the odds ratio ρ [21]. This index measures the abundance of dinucleotides relative to what would be expected from the random union of mononucleotides [21] and the value 1 is expected when no bias is observed.

Both theoretical and empirical studies have indicated that if a given dinucleotide has a ρ index value ≤0.78 then this dinucleotide is significantly underrepresented (suppressed) whereas values ≥1.23 indicates over-representation [21]. For coding sequences the average dinucleotide bias was calculated separately for the three reading frames. Interestingly, in both *Arabidopsis* and rice there was a pervasive suppression of CG and TA dinucleotides in all three reading frames (table 3).

**Table 3:** Dinucleotide biases observed in the coding sequences of *Arabisopsis* and *Oryza*.

	*Arabidopsis*			*Oryza*		
**Dinucleotide**	**1_2**	**2_3**	**3_1**	**1_2**	**2_3**	**3_1**
CG	0.71^†^	0.56^†^	0.64^†^	0.75^†^	0.64^†^	0.71^†^
TA	0.42^†^	0.67^†^	0.69^†^	0.47^†^	0.67^†^	0.68^†^
CA	0.97	1.23*	**1.30**	0.96	**1.43**	**1.27**
TG	0.82	**1.18**	**1.21**	0.89	**1.16**	**1.33**
AA	**1.16**	**1.08**	**1.03**	1.65*	**1.03**	**1.10**
AC	0.75^†^	0.96	0.90	0.74	0.89	0.86
GA	1.24*	**1.09**	**1.22**	**1.19**	0.90	**1.04**
GT	0.73^†^	0.94	0.92	0.72^†^	0.89	0.89
TC	1.47*	**1.19**	**1.12**	1.42*	**1.08**	0.92
TT	1.39*	**1.03**	1.00	1.29*	**1.03**	0.96
CC	**1.09**	0.83	0.95	1.00	0.88	**1.03**
AG	**1.16**	**1.15**	**1.08**	**1.12**	**1.12**	0.96
AT	0.93	0.87	0.93	0.98	**1.00**	**1.13**
GG	0.88	**1.10**	0.88	**1.13**	**1.01**	0.92
GC	0.88	0.99	0.98	0.99	**1.21**	**1.16**
CT	**1.14**	1.20	**1.22**	1.23*	**1.08**	**1.08**

*Oryza* (bold  =  overrepresented, plain  =  underrepresented, * = significantely over-represented, ^†^.  =  significantely under-represented).

These tendencies were confirmed for introns of both species with the only difference that the average CG under-representation in introns was stronger than in exons while the opposite was found for TA (table 4) Other interesting differences were revealed by the analysis of dinucleotide graphs: while TA suppression was almost constant, CG under-representation increased proceeding along the direction of transcription (for a complete picture of the observed trends as a function of position see figures S9, S10, S11, S12, S13, S14, S15, S16).

**Table 4:** Average dinucleotide biases in introns and exons of *Arabidopsis* and *Oryza* (bold  =  overrepresented, plain  =  underrepresented, * = significantely over-represented, ^†^.  =  significantely under-represented).

	*Arabidopsis*		*Oryza*	
Dinucleotide	Intron	Exon	Intron	Exon
AA	**1.15**	**1.12**	**1.14**	**1.10**
AG	**1.03**	**1.11**	0.98	1.03
AC	0.93	0.88	0.89	0.85
AT	0.91	0.88	0.98	**1.04**
GA	**1.14**	1.24*	**1.01**	**1.11**
GG	0.95	0.96	**1.04**	**0.96**
GC	0.94	0.91	**1.13**	**1.11**
GT	0.95	0.86	0.91	0.84
CA	**1.13**	**1.10**	**1.18**	**1.12**
CG	0.56^†^	0.70^†^	0.59^†^	0.86
CC	0.97	0.96	**1.09**	0.96
CT	**1.11**	**1.16**	**1.03**	**1.06**
TA	0.79	0.63^†^	0.80	0.67^†^
TG	**1.18**	**1.13**	**1.20**	**1.11**
TC	**1.09**	**1.21**	0.96	**1.08**
TT	**1.04**	**1.11**	**1.06**	**1.10**

TG and AC over-representation in introns can be related to CG suppression under the the methylation -deamination- mutation scenario (i.e. CpG islands tend to be easily methylated at the Cytosine residue with a consequent increase in the rate of C -> T mutation). However TG and AC biases in CDS did not mirror accurately the pattern of CG suppression: TG over-representation at 2_3 was lower than at 3_1 in spite of the higher suppression of CG at frames 2_3 than 3_1. A group of five dinucleotides, AA, TC, GT, TT, TC were biased in frame 1_2 only. Such a pattern was probably related to the requirements of specific aminoacids, a hypothesis that was supported also by the virtual absence of bias in intron sequences.

Another group of five dinucleotide (CC, AG, AT, GG, GC,CT) showed no significant bias in all the three reading frames of cds and in introns.

The last two dinucleotides GA and CT showed a different bias in the two species. GA was biased in frames 1_2 and 2_3 of *Arabidopsis* cds but completely unbiased in rice. CT was overrepresented in frames 2_3 and 3_1 in *Arabidopsis* and to lower extent in frame 1_2 while the opposite trend was showed in rice, biased in frame 1_2 but not in frames 2_3 and 3_1. These features are likely to reflect specific differences in the global dinucleotide signature of the two species or differences in codon usage. As a matter of fact, both dinucleotide are slightly overrepresented in *Arabidopsis* introns but are unbiased in those of rice.

### Tri-nucleotide trends

The complete lists of average values of the index of tri nucleotide bias for both *Arabidopsis* and rice coding sequences and introns are reported in tables S2, S3. On average, the bias of trinucleotides was low if compared to that of either mono and dinucleotides This was particulary evident for trinucleotide in introns of both species. The most over-represented trinucleotide showed a γ index of 1.14 in rice and 1.12 in *Arabidopsis* while the most suppressed showed values of 0,878 (CAG) in *Arabidopsis* and 0,930 (AAG) in rice (table 5). In coding sequences the distribution of the bias was highly dependent on the class of the trinucleotide relatively to the reading frame. In fact, the values of the correlations between γ_XYZ_ of the same frame in the two species were always higher than those between γ_XYZ_ with different frames within either species (Table 6).

**Table 5:** Trinucleotide biases in introns of *Arabidopsis* and *Oryza sativa*.

Arabidopsis		Rice	
trinucleotides		trinucleotides	
CCG	**1.14**	TAG	**1.12**
CCA	**1.11**	GAT	**1.12**
GAA	**1.11**	GAG	**1.09**
TAG	**1.1**	GCC	**1.08**
GAG	**1.08**	CCG	**1.06**
CGC	0.81	AAG	0.92
GCG	0.87	GGT	0.94
CAG	0.87	TTG	0.94
CCT	0.88	CAG	0.94
AGG	0.92	GTC	0.94

(*Oryza* (bold  =  overrepresented, plain  =  underrepresented).

**Table 6:** Trinucleotide biases correlations between *Arabidopsis thaliana* and *Oryza sativa* (p<0.01).

	1_2_3(Ara)	2_3_1(Ara)	3_2_1(Ara)	1_2_3(Rice)	2_3_1(Rice)
**2_3_1(Ara)**	0.6699	–			
**3_2_1(Ara)**	0.5245	0.6943	–		
**1_2_3(Rice)**	**0.9219**	0.6415	0.4396	–	
**2_3_1(Rice)**	0.4756	**0.8034**	0.4819	0.551	–
**3_2_1(Rice)**	0.4212	0.6051	**0.9001**	0.3759	0.5378

### Compositional gradients of bryophytes coding sequences

**Figure 4:**
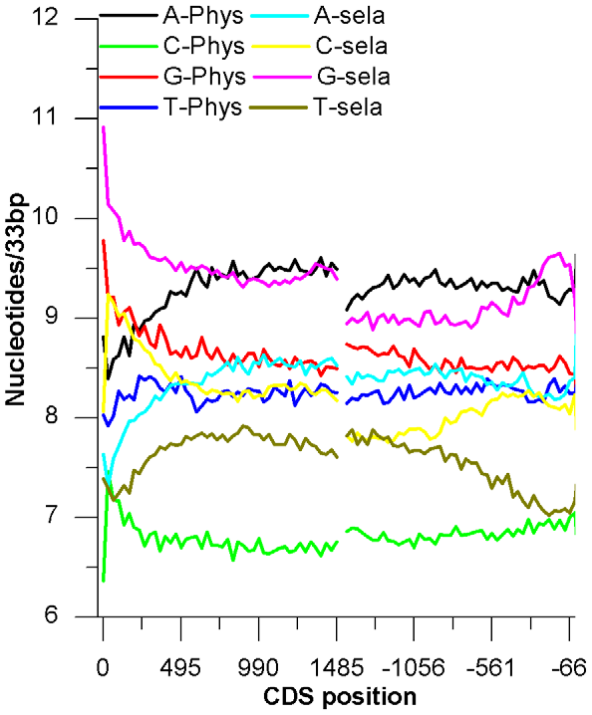
Mononucleotide content of bryophytes CDS. Overall mononucleotides content as function of position in *Physcomitrella* and *Selaginella* coding sequences and averaged over all sequences in the dataset with a 33 bp adiacent window.

The presence of genic compositional gradients was traced back in the evolution of green plants by analysing the coding sequences of bryophytes. Mononucleotide contents were studied as function of position along *Physcomitrella patens* [22] and *Selaginella moellendoerfi* [23] coding sequences. As shown in figure 4 both these bryophyte species showed compositional gradients that closely resembled to those observed for higher plants. As reported above, the most distinctive features between monocots and dicots species was the guanine trends of cds which appeared concave in monocots and convex in dicots. Both bryophytes showed a monocot type guanine trend, firmly decreasing in the first part of the sequence while weakly increasing toward the 3′end. The adenine trends were increasing in both bryophytes whereas cytosines depicted an opposite decreasing trend.The tymine trends marked an evident dissimilarity, with the *S. moellendoerfi* T content decreasing more sharply toward the 3′end. As reported for higher plant species also the bryophyte profiles calculated on datasets partitioned based on cds length or overall GC content produced pictures that were compatible with those obtained on the whole dataset (Figures S17, S18, S19, S20). Similarly, the resolution of the mononucleotide compositional profiles above in the contributions attributable to the first, second and third codon base confirmed the picture already described for higher plant species with the third base explaining most of the variation (Figures S21, S22).

Dinucleotide bias is considered a salient feature of the genomic signature of a species [3]. The pattern of cds dinucleotide bias was similar for *Physcomitrella patens* and *Selaginella moellendoeri* (see Table 7). We identified most of the hallmarks of higher plant dinucleotide bias in bryophyte cds'. For example the highly suppressed TA and CG content were also significantly underrepresented in bryophyte coding sequences. For a complete picture of dinucleotide bias as function of position in *P.patens* cds see figures S23, S24, S25. On average, the dinucleotide bias of the bryophytes coding sequences was lower than that of higher plants (Figures S26, S27). This observation was reproduced for all three reading fames (1_2,2_3and 3_1). A similar conclusion was drawn for trinucleotide bias (Table S4). The absolute level of bias was low in both *P patens* and *S. moellendorfii* suggesting that mononucleotides and dinucleotides account for most compositional heterogeneity of bryophyte coding sequence. Comparative analysis with higher plants revealed that for all three reading frames the trinucleotide bias of bryophyte was lower than that of both rice and Arabidopsis (S26–S27).

**Table 7:** Average dinucleotide biases in CDS of Physcomitrella and Selaginella (bold  =  overrepresented, plain  =  underrepresented, * = significantely over-represented, ^†^.  =  significantely under-represented).

	Physcomytrella				Selaginella			
**Dinucleotide**	**All**	**1_2**	**2_3**	**3_1**	**All**	**1_2**	**2_3**	**3_1**
AA	**1.06**	**1.13**	**1.09**	**1.00**	**1.07**	**1.14**	**1.09**	**1.05**
AG	**1.04**	**1.05**	**1.04**	**1.08**	**1.09**	**1.14**	**1.11**	**1.11**
AC	0.87	0.83	0.94	0.88	0.86	0.79	0.93	0.83
AT	0.96	0.97	0.93	0.99	0.92	0.94	0.88	0.94
GA	**1.15**	**1.21**	**1.04**	**1.09**	**1.19**	**1.20**	**1.19**	**1.08**
GG	0.96	1.08	0.99	0.95	0.92	1.04	0.89	0.94
GC	**1.05**	0.97	**1.14**	**1.07**	**1.07**	**1.05**	**1.15**	**1.12**
GT	0.80	0.74^†^	0.89	0.88	0.78	0.72^†^	0.83	0.85
CA	**1.17**	**1.01**	1.25*	1.34*	**1.12**	**0.95**	1.24*	1.25*
CG	0.74^†^	0.95	0.67^†^	0.73^†^	0.87	0.83	0.86	0.88
CC	0.94	**1.00**	0.94	0.90	0.82	0.91	0.83	0.77^†^
CT	**1.09**	**1.03**	**1.12**	**1.09**	**1.17**	1.25*	**1.17**	**1.16**
TA	0.60^†^	0.43^†^	0.66^†^	0.71^†^	0.52^†^	0.48^†^	0.58^†^	0.57^†^
TG	**1.17**	0.85	1.24*	**1.15**	**1.10**	0.94	**1.07**	**1.13**
TC	**1.11**	1.32*	**1.03**	**1.09**	**1.21**	1.32*	**1.12**	1.24*
TT	**1.15**	1.50*	**1.06**	**1.07**	**1.14**	1.33*	**1.11**	**1.09**

## Discussion

Compositional heterogeneity may be described at many genomic scales. In the present paper we used an approach based on ensemble averages to compile descriptions of compositional trends in transcribed genic sequences of several eudicots and monocots species. Two provocative considerations were evoked from the first glance of the graphs. First, the shapes of the trends were different between monocots and eudicots but surprisingly uniform within the two evolutionary clades. Second, the variations observed in the first part of the sequences petered out toward the 3'end of the genes.

Both deductions are in agreement with previous findings reported by Wong and co-worker in a study on the G+C content of genic regions of several monocot and eudicot species [17]. However, that study did not detail on differences between single bases. For example, the guanine content decreases firmly in monocots and increases, although weakly, in eudicots. Thymine increases in the first part of the cds in both eudicots and monocots and then decreases in the eudicots but not in monocots. Before starting to speculate on the emergence of these differences we cannot elude a fundamental question: what proportion of all single genes trends fit to the ensemble models?

In answering this question it should be kept in mind that most of the factors influencing single sequence's compositions are highly variable and consequently the assignment of single gene trends to stringently defined categories can be a hardly tractable goal. On the light of these considerations, we concluded that the definition of the exact proportion of genes fitting to the ensemble models should first leave place to more qualitative assessments. The most informative part of ensemble graphs were approximated to linear models and then single gene trends were studied for their consistency with these models. With this experimental setup we could demonstrate that the i) shape of compositional trends is independent from sequence length, genomic orientation or overall GC content and ii) that most of the single gene parameters distributions are in agreement with the expectations based on the models described by the ensemble approach. Inherently we should emphasize that the breakpoint distributions covered a wide range of values and that the classes of highest frequency did not obviously correspond to what foreseen by the ensemble model. Whether this is due to the high variability of the evolutionary forces shaping the gradients or is the results of factors of other nature such as for example the genomic position of the genes is still to be determined. Our analyses of bryophytes coding sequences suggested that these compositional gradients were already emerged at the time of land conquest by green plants. Based on these results we suggest that the ensemble graphs can be considered as *master* models for plant genes.

With these propositions in mind we can move to the next question: what are the evolutionary forces responsible for these compositional arrangements?

A caveat of all further considerations should be the polarity of the trends which may help in limiting the range of choices. Indeed mutational or selective forces acting at DNA level are expected to exert an effect in opposite direction in genes with either forward or reverse orientation. The trends calculated on genic sequences grouped based on their orientation in the genome did not reveal these types of evidences. Because the polarity of the trends was in the same direction of transcription/translation we will restrict our discussion to forces related to these two basic cellular processes.

Observations conducted in other systems may help in constructing insightful analogies, Eyre and Walker has shown that the first portion of *E.coli* genes is compositionally different from the remaining part [5]. The explanations proposed envisaged selection for i) the mRNA secondary structure near the ribosome binding site and ii) the use of suboptimal codons to regulate gene expression. Evident biases near the translation initiation codon of coding sequences of seven eukaryote genomes, including *Arabidopsis* and *Oryza*, has been reported by Niimura et al and were explained in terms of selection for efficiency of translation initiation [16]. Our data featured a strong variations in base composition at the beginning of CDS and therefore do not rule out the involvement of this type of selection in the generation of the gradients. But if so, to account for the difference in compositional features between these two species, we should hypothesize that the target of this type of selection is rather different in monocots and eudicots. Indeed, Gu et al have recently represented a universal trend of reduced mRNA stability near the translation sites in 340 species including eukaryotes and prokaryotes [24]. Very interesting rice and *Arabidopsis* genes marked a contrasting behavior in this respect. The sequences near the translation starting codon showed a thermodynamic stability slightly higher than that expected by chance in rice genes and moderately reduced in *Arabidopsis*. Furthermore, while in most species, the genes with higher codon bias had lower mRNA stability at their 5' end, highly biased rice genes showed very stable mRNA at the 5' end and the opposite was observed for the low biased genes. This finding supports the hypothesis that the selection near the translation starting codon have singular feature in rice and perhaps may be used as argument to explain some of the differences found in the first portion of *Arabidopsis* and rice graphs. However we question whether a different deal of selection for translation initiation efficiency may generates differences distributed along a quite large portion of genes. Moreover we cannot figure out in such context the significance of the gradients observed in introns.

De-Rose-Wilson et al. [25] have recently proposed that transcription related mutation (TCR) contributes significantly to rate differences between intergenic and transcribed sequence in *Arabidopsis* genome. Similar conclusion may be reached considering the pattern of strand asymmetry in intergenic and genic sequences in rice (our unpublished results). These findings coupled to recent insights on the transcription coupled repair process may provide ground for explanations of some of the gradients. Experiments conducted in animal systems have indicated that the speed of the transcription coupled repair process may be dependent on the position of the lesion within the transcribed sequence a feature that may be even related to differences in the subunits involved. It is therefore possible that gradients in speed or even fidelity of TCR may contribute significantly to the establishment of compositional gradients within genic regions.

The pattern of dinucleotide bias in coding sequence and in introns disclosed other important compositional features of plant genes. Studies carried out on coding or non coding sequence as well as at whole genome level [26] have documented, for example, a pervasive under-representation of the CG and TA dinucleotides in plants. This study confirmed these tendencies also in bryophyte coding sequences and underlined new feature of this phenomenon. For example CG suppression was more severe in introns then in exon probably reflecting a higher mutation rate and/or low selective constrains in non coding genic sequences. Moreover the suppression increased proceeding along the direction of transcription in both monocots and eudicots. This effect was only partially mirrored by an over-representation of TG suggesting that other causes than the classical methylation-deamination-mutation scenario could explain the dependence of CG underepresentaion with position in gene sequences. It is known that CG dinucleotides posses the highest thermodynamic stacking energy; the variation of CG suppression along the direction of transcription may therefore aid DNA untwisting during transcription. Other dinucleotides showed different biases in the 1_2 compared to 2_3 and 3_2 reading frames of cds likely reflecting different amino-acid or codon usage. The overall trinucleotide bias was on average quite low in introns confirming the hypothesis that the forces maintaining structural features of DNA act prevalently at level of mono or dinucleotides. Interestingly also the bias at the reading frame 1_2_3 of most trinucleotides was rather low suggesting that a non trivial part of the codon usage bias of plant genes can be explained by compositional features related to DNA structural properties.

In conclusion, we presented a detailed investigation of the compositional features of genic sequence which identified master models of compositional trends. These models can be considered as a “genomic reference” to describe the compositional feature of groups of genes related for some structural or functional features. We are confident that some of these contrasts will find robust associations with some of the compositional features highlighted in this study and therefore may be instrumental in identifying casual links.

## Supporting Information

Figure S1 Mononucleotide content of *Arabidopsis* as function of position starting from the first codon and proceeding along translation direction and averaged over all sequences in each dataset with an adjacent window of 33 bp. The sequences were partitioned based on their length in seven dataset.
(TIF)

Figure S2 Mononucleotide content of *Oryza* as function of position starting from the first codon and proceeding along translation direction and averaged over all sequences in each dataset with an adjacent window of 33 bp. The sequences were partitioned based on their length in seven dataset.
(TIF)

Figure S3 Overall mononucleotide compositional trends of *Arabidopsis* coding sequences as a function of position and averaged over all sequences of each dataset with an adjacent window of 33 bp. The trends are calculated from the dataset including cds of genes with forward (black) or reverse (red) orientation.
(TIF)

Figure S4 Overall mononucleotide compositional trends of Oryza coding sequences as a function of position and averaged over all sequences of each dataset with an adjacent window of 33 bp. The trends are calculated from the dataset including cds of genes with forward (black) or reverse (red) orientation.
(TIF)

Figure S5 Overall mononucleotide compositional trends of *Arabidopsis* coding sequences as a function of position and averaged over all sequences of each dataset with an adjacent window of 33 bp. The dataset “low” included sequence with a G+C content < = 0.40. The dataset medium included sequences with a G+C content >0.40 and < = 0.50. Sequence with a G+C >0.50 were included in the dataset “high”.
(TIF)

Figure S6 Overall mononucleotide compositional trends of *Oryza* coding sequences as a function of position and averaged over all sequences of each dataset with an adjacent window of 33 bp. The dataset “low” included sequence with a G+C content < = 0.40. The dataset medium included sequences with a G+C content >0.40 and < = 0.50. Sequence with a G+C >0.5 were included in the dataset “high”.
(TIF)

Figure S7 Break point distribution for the 4 bases of *Arabidopsis* and *Oryza* (BPc  =  consensus break point).
(TIF)

Figure S8 Ensemble graphs of 5' and 3' UTR untranslated sequences. The adjacent window was of 33 bp.
(TIF)

Figure S9 Dinucleotide content of the first 2 kb of *Arabidopsis* cds. The dinucleotide were calculated taking into account the first and second position of each codon.
(TIF)

Figure S10 Dinucleotide (2_3) content of the first 2 kb of *Arabidopsis* cds. The dinucleotide were calculated taking into account the second and third position of each codon.
(TIF)

Figure S11 Dinucleotide content of the first 2 kb of *Arabidopsis* cds. The dinucleotide were calculated taking into account the third position of a codon and the first of the subsequent codon.
(TIF)

Figure S12 Dinucleotide content of the first 2 kb of *Oryza* cds. The dinucleotide were calculated taking into account the first and second position of each codon.
(TIF)

Figure S13 Dinucleotide content of the first 2 kb of *Oryza* cds. The dinucleotide content were calculated taking into account the second and third position of each codon.
(TIF)

Figure S14 Dinucleotide content of the first 2 kb of *Oryza* cds. The dinucleotide content were calculated taking into account the third position of a codon and the first of the subsequent codon.
(TIF)

Figure S15 Dinucleotide content of the first 2.5 kb of *Arabidopsis* introns.
(TIF)

Figure S16 Dinucleotide content of the first 2.5 kb of *Oryza* introns.
(TIF)

Figure S17 Mononucleotide content of *Physcomitrella* as function of position starting from the first codon and proceeding along translation direction and averaged over all sequences in each dataset with an adjacent window of 33 bp. The sequences were partitioned based on their length in seven dataset.
(TIF)

Figure S18 Mononucleotide content of *Selaginella* as function of position starting from the first codon and proceeding along translation direction and averaged over all sequences in each dataset with an adjacent window of 33 bp. The sequences were partitioned based on their length in seven dataset.
(TIF)

Figure S19 Overall mononucleotide compositional trends of *Physcomitrella* coding sequences as a function of position and averaged over all sequences of each dataset with an adjacent window of 33 bp. The dataset “low” included sequence with a G+C content < = 0.40. The dataset medium included sequences with a G+C content >0.40 and < = 0.50. Sequence with a G+C >0.5 were included in the dataset “high”.
(TIF)

Figure S20 Overall mononucleotide compositional trends of *Selaginella* coding sequences as a function of position and averaged over all sequences of each dataset with an adjacent window of 33 bp. The dataset “low” included sequence with a G+C content < = 0.40. The dataset medium included sequences with a G+C content >0.40 and < = 0.50. Sequence with a G+C >0.5 were included in the dataset “high”.
(TIF)

Figure S21 Overall first, second and third codon nucleotide content as function of position in *Physcomitrella*.
(TIF)

Figure S22 Overall first, second and third codon nucleotide content as function of position in *Selaginella.*
(TIF)

Figure S23 Dinucleotide content of the first 2 kb of *Physcomitrella* cds. The dinucleotide were calculated taking into account the first and second position of each codon.
(TIF)

Figure S24 Dinucleotide content of the first 2 kb of *Physcomitrella* cds. The dinucleotide content were calculated taking into account the second and third position of each codon.
(TIF)

Figure S25 Dinucleotide content of the first 2 kb of *Physcomitrella* cds. The dinucleotide content were calculated taking into account the third position of a codon and the first of the subsequent codon.
(TIF)

Figure S26 Linear fitting of the trinucleotide biases of *Oryza*, *Arabidopsis*, *Physcomytrella* and *Selaginella.*
(TIF)

Figure S27 Linear fitting of the dinucleotide biases of *Oryza*, *Arabidopsis*, *Physcomytrella* and *Selaginella.*
(TIF)

Table S1 (**a**) Break points and slopes of the ensemble graphs for Oryza sativa. Slopes are expressed in bp/Kbp. (b) Percentage of significant fitting to the segmented and linear regression for *Ozyza sativa*.
(DOC)

Table S2 (**a**) Average trinucleotide bias of Arabidopsis coding sequences. The trinucleotide contents were calculated for each window position and averaged over all sequences longer than 2 kb using a window of 99 bp. The γ index were calculated according to Karlin [2]. (b) Average trinucleotide bias of Oryza coding sequences. The trinucleotide contents were calculated for each windoe position and averaged over all sequences longer than 2 kb using a window of 99 bp. The γ index were calculated according to Karlin [2].
(DOC)

Table S3 Average trinucleotide bias of intron sequences. The trinucleotide contents were calculated for each window position and averaged over all sequences.
(DOC)

Table S4 Average trinucleotide bias of *Physcomytrella* and *Selaginella* coding sequences. The trinucleotide contents were calculated for each windoe position and averaged over all sequences longer than 2 kb using a window of 99 bp. The γ index were calculated according to Karlin [2].
(DOC)
