# Supplementary figures and images for: Spatial Analyses of Mono, Di and Trinucleotide Trends in Plant Genes
Source: PLoS One. 2011 Aug 1;6(8):e22855. doi: 10.1371/journal.pone.0022855 (PMC3148226; doi:10.1371/journal.pone.0022855)

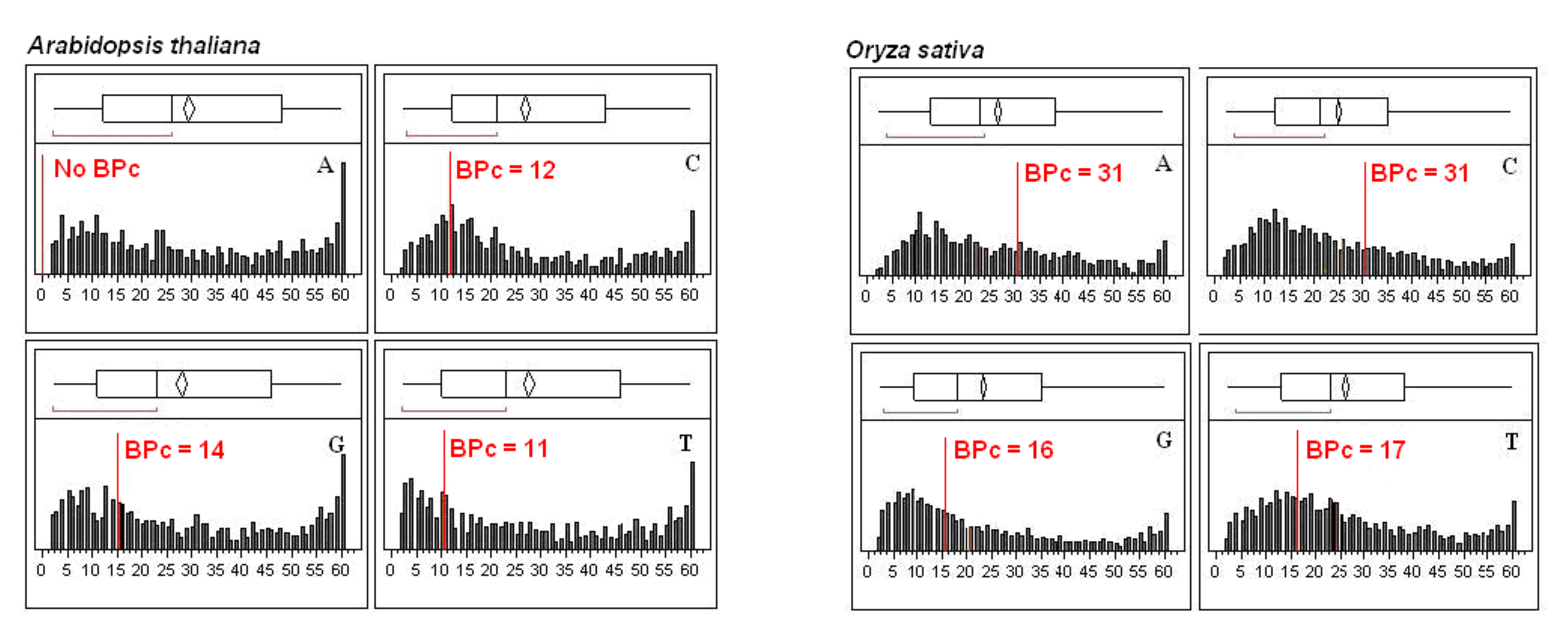

Supplement: Figure S7 — Break point distribution for the 4 bases of Arabidopsis and Oryza (BPc = consensus break point). (TIF) [file pone.0022855.s007.tif]

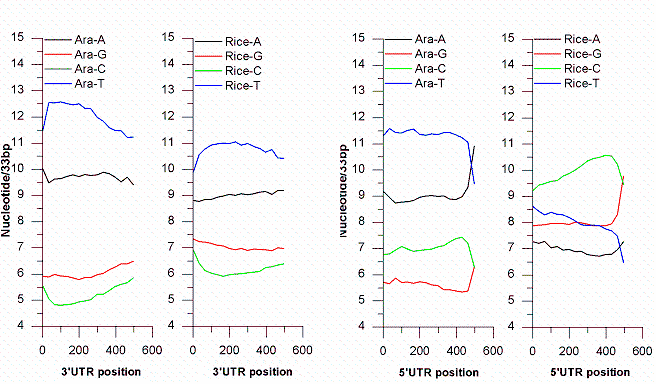

Supplement: Figure S8 — Ensemble graphs of 5' and 3' UTR untranslated sequences. The adjacent window was of 33 bp. (TIF) [file pone.0022855.s008.tif]

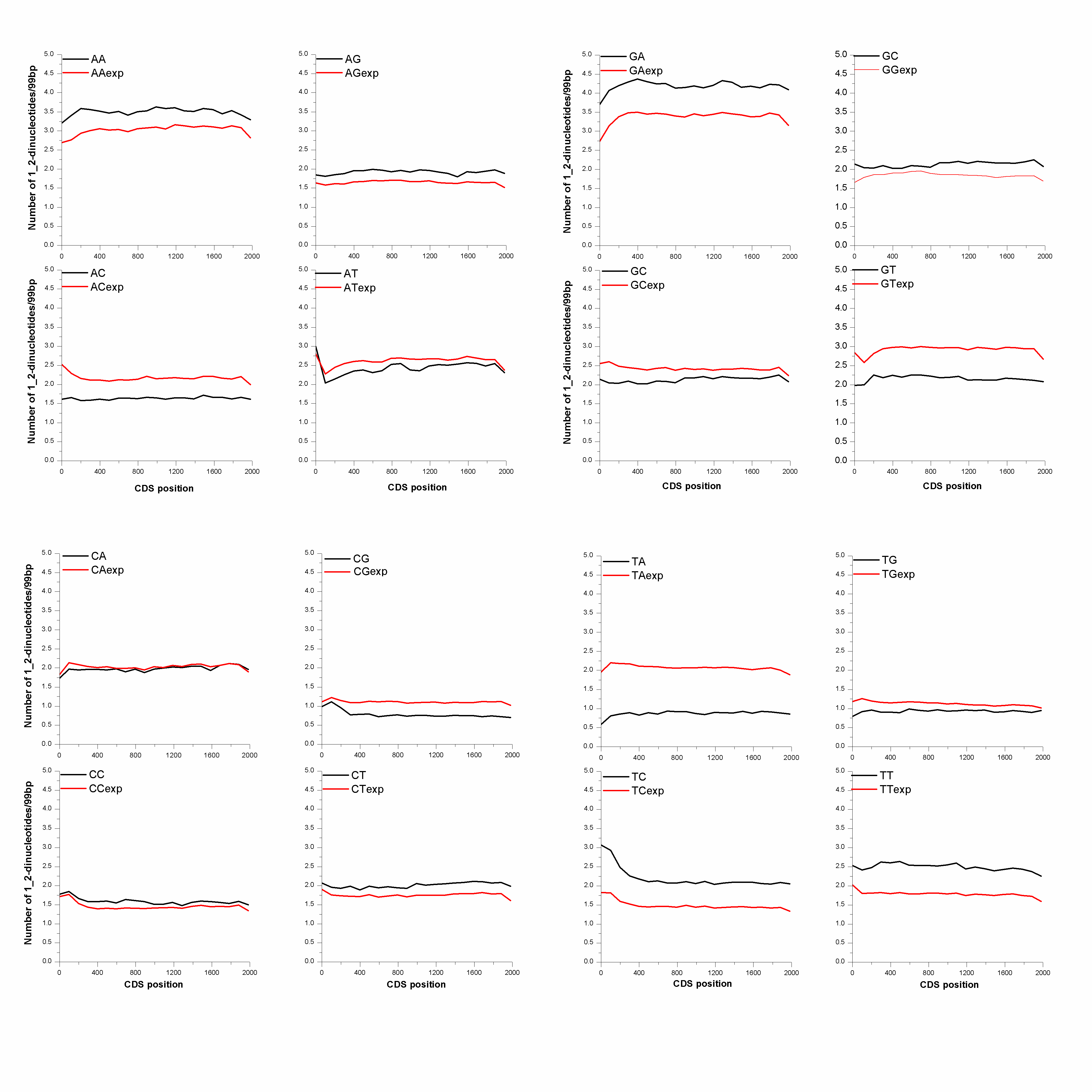

Supplement: Figure S9 — Dinucleotide content of the first 2 kb of Arabidopsis cds. The dinucleotide were calculated taking into account the first and second position of each codon. (TIF) [file pone.0022855.s009.tif]

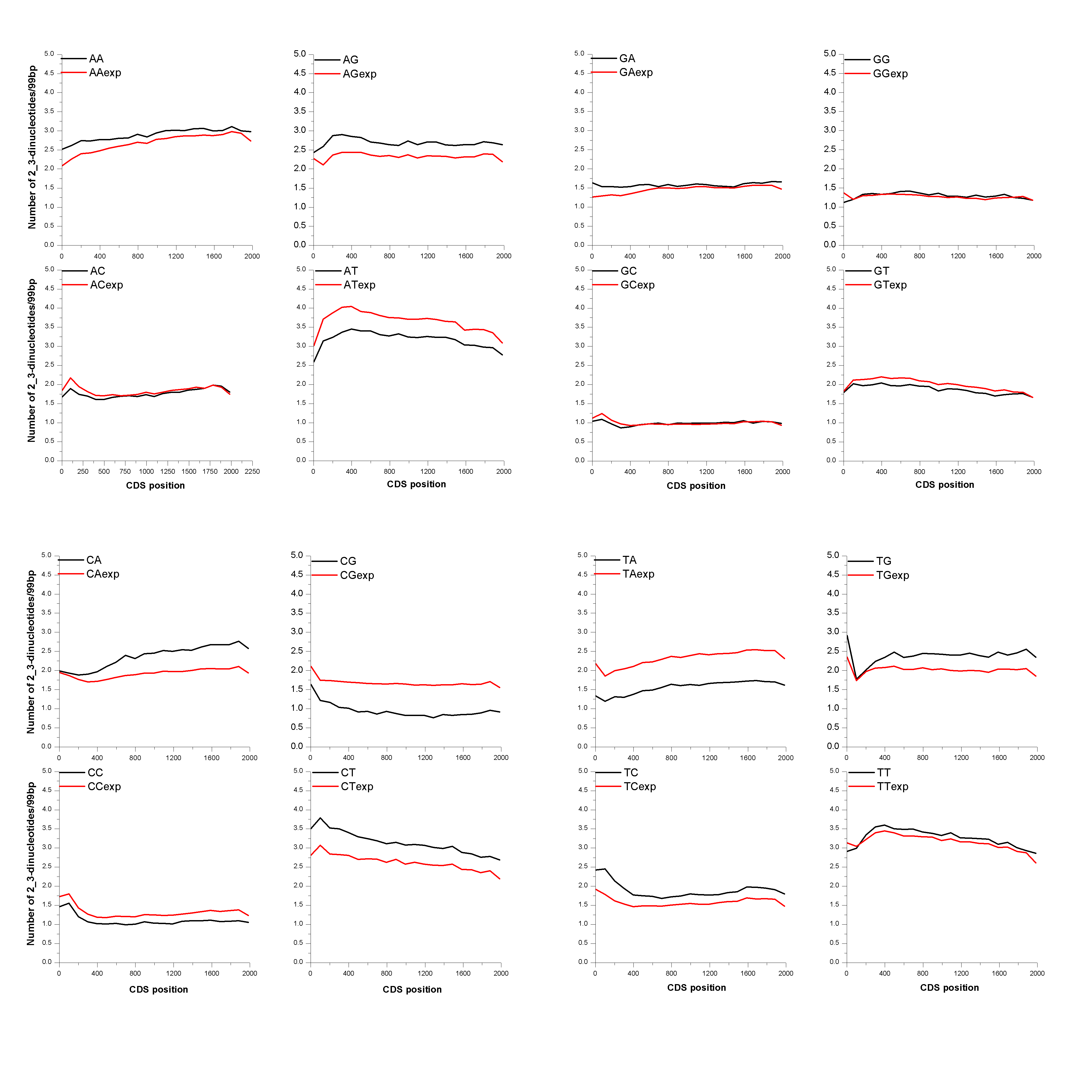

Supplement: Figure S10 — Dinucleotide (2_3) content of the first 2 kb of Arabidopsis cds. The dinucleotide were calculated taking into account the second and third position of each codon. (TIF) [file pone.0022855.s010.tif]

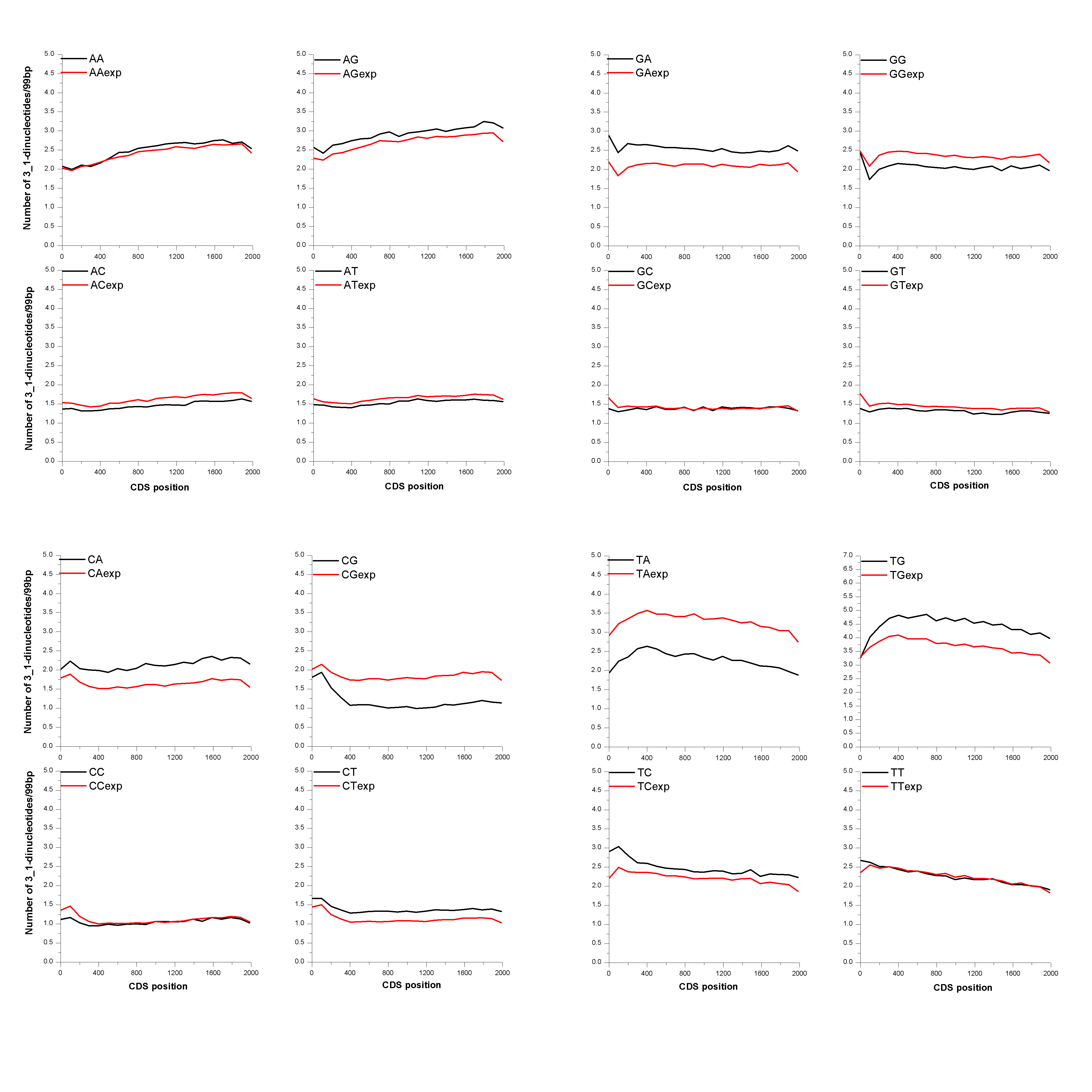

Supplement: Figure S11 — Dinucleotide content of the first 2 kb of Arabidopsis cds. The dinucleotide were calculated taking into account the third position of a codon and the first of the subsequent codon. (TIF) [file pone.0022855.s011.tif]

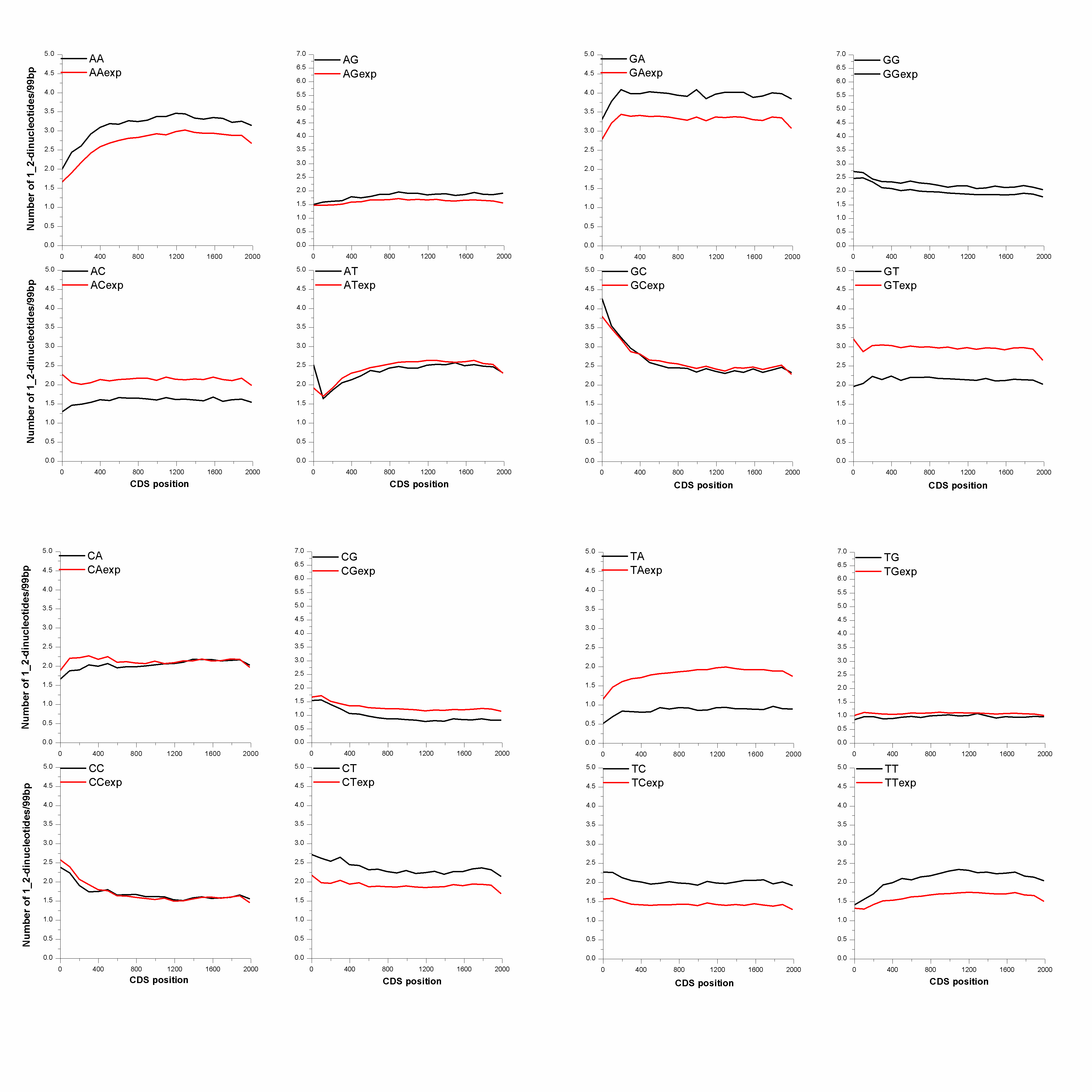

Supplement: Figure S12 — Dinucleotide content of the first 2 kb of Oryza cds. The dinucleotide were calculated taking into account the first and second position of each codon. (TIF) [file pone.0022855.s012.tif]

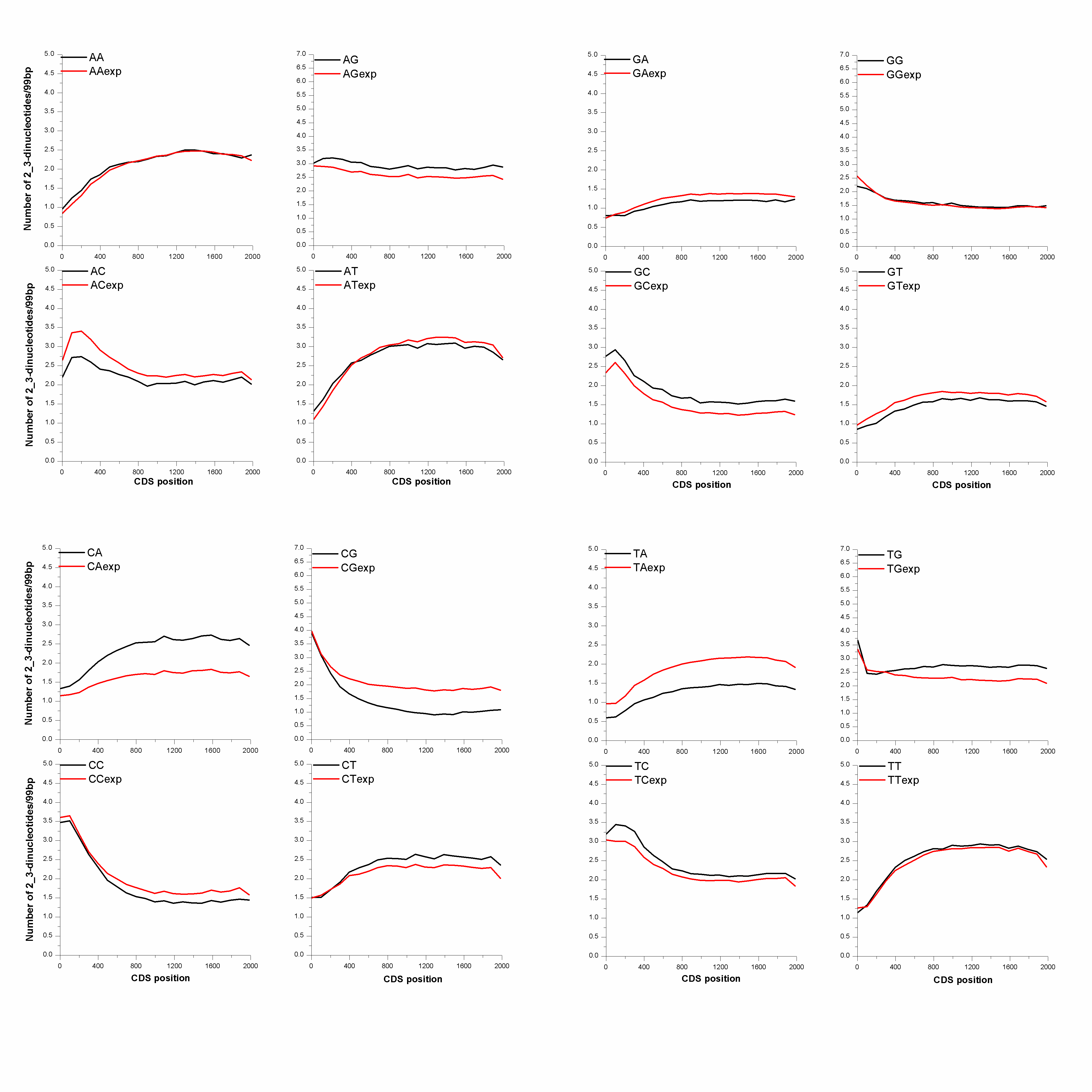

Supplement: Figure S13 — Dinucleotide content of the first 2 kb of Oryza cds. The dinucleotide content were calculated taking into account the second and third position of each codon. (TIF) [file pone.0022855.s013.tif]

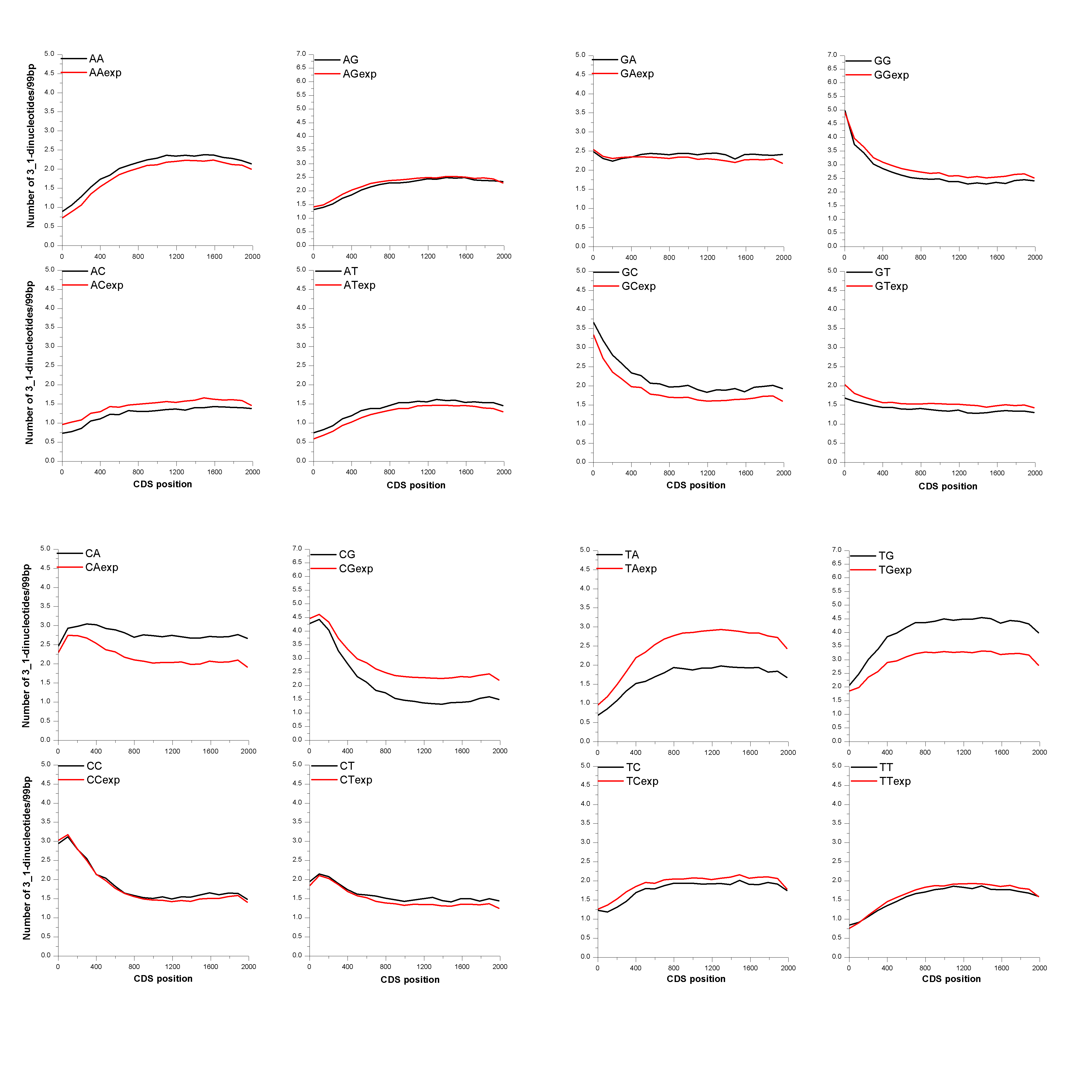

Supplement: Figure S14 — Dinucleotide content of the first 2 kb of Oryza cds. The dinucleotide content were calculated taking into account the third position of a codon and the first of the subsequent codon. (TIF) [file pone.0022855.s014.tif]

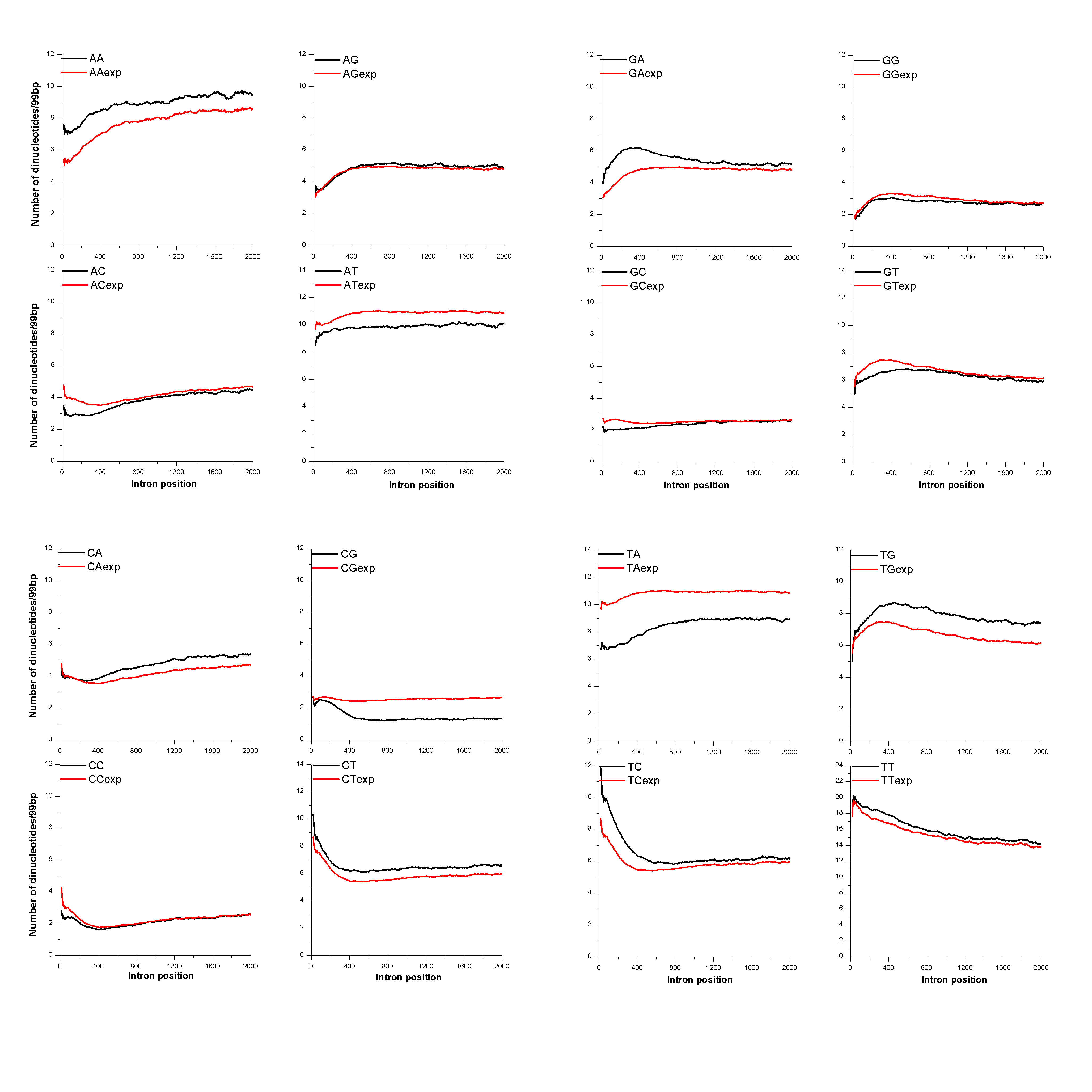

Supplement: Figure S15 — Dinucleotide content of the first 2.5 kb of Arabidopsis introns. (TIF) [file pone.0022855.s015.tif]

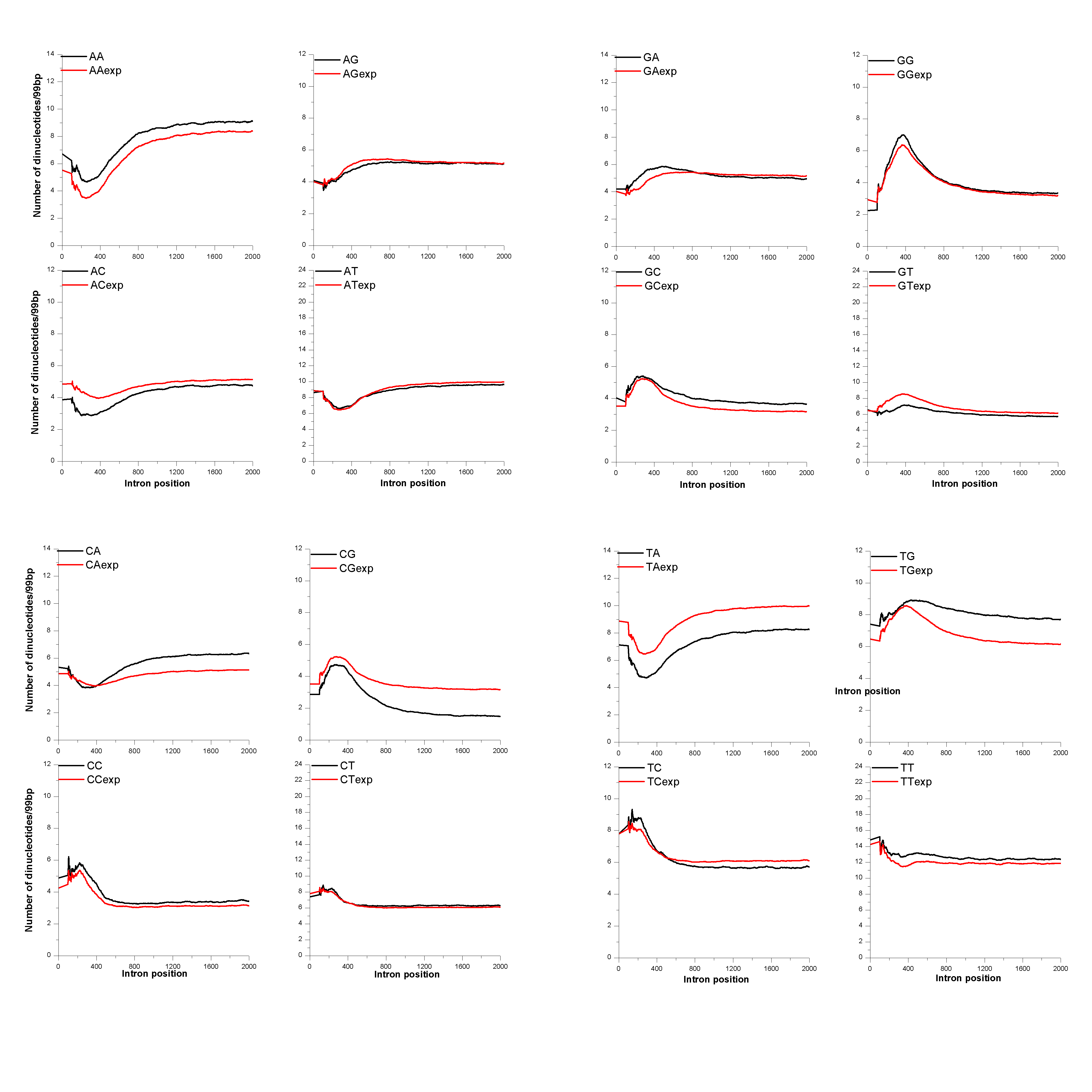

Supplement: Figure S16 — Dinucleotide content of the first 2.5 kb of Oryza introns. (TIF) [file pone.0022855.s016.tif]

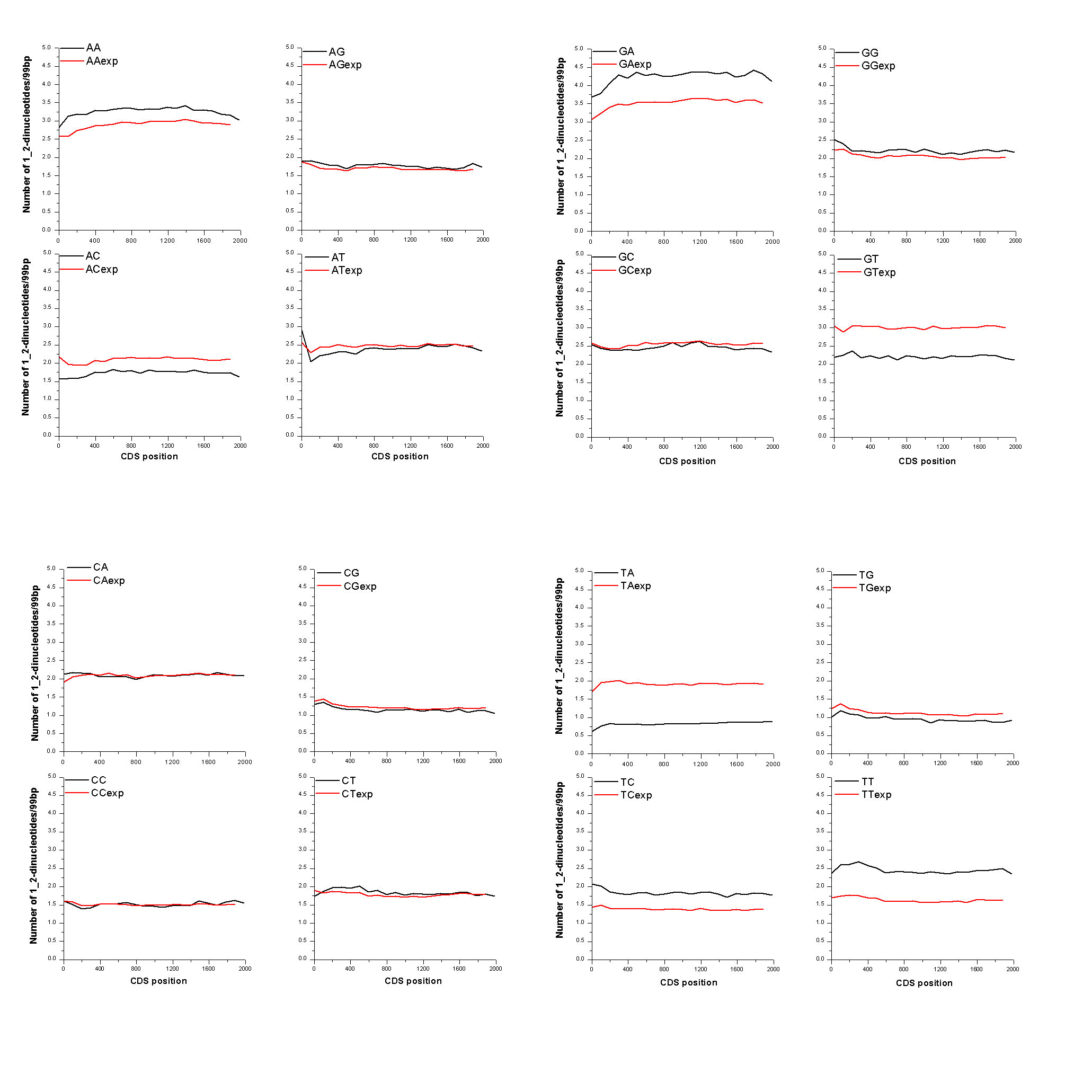

Supplement: Figure S23 — Dinucleotide content of the first 2 kb of Physcomitrella cds. The dinucleotide were calculated taking into account the first and second position of each codon. (TIF) [file pone.0022855.s023.tif]

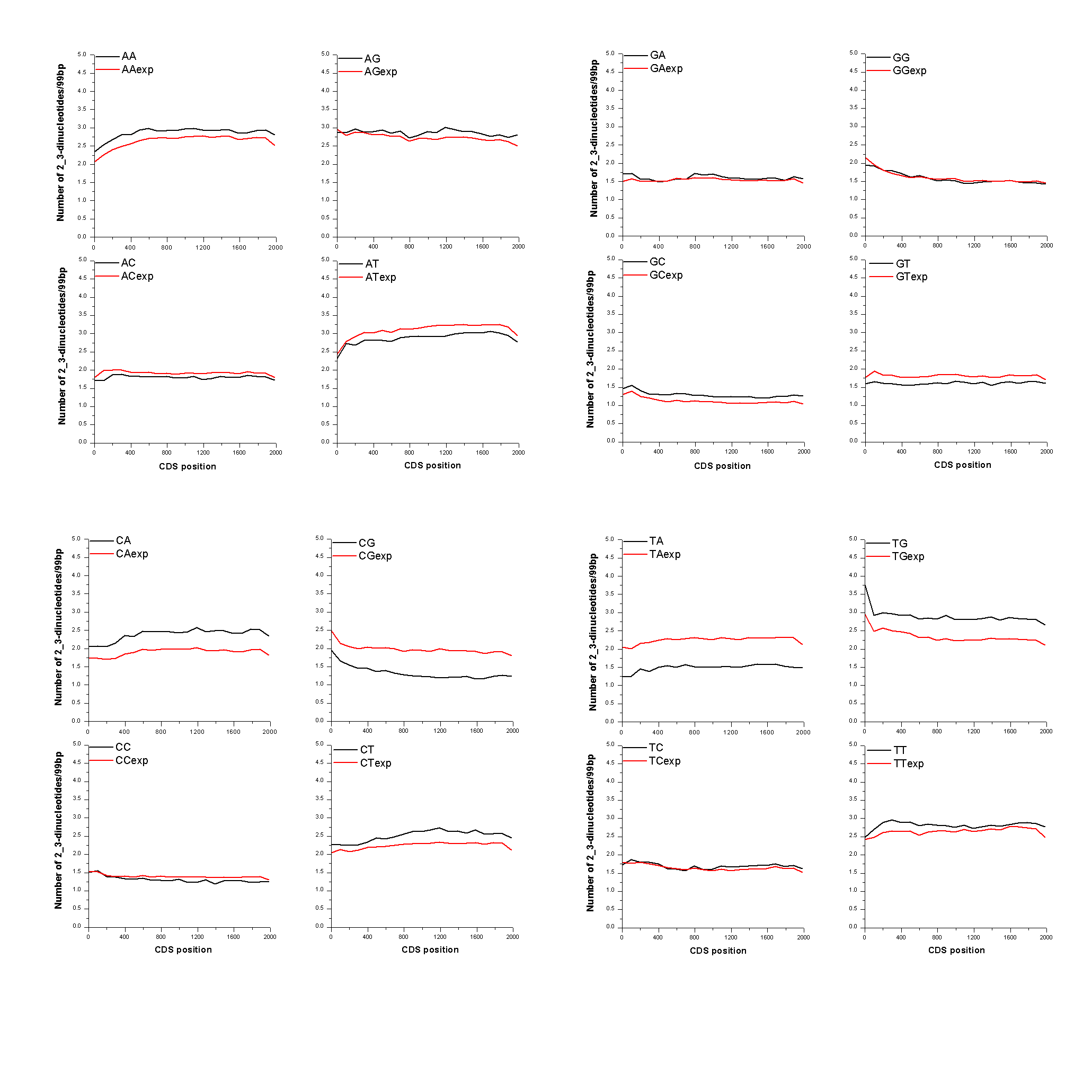

Supplement: Figure S24 — Dinucleotide content of the first 2 kb of Physcomitrella cds. The dinucleotide content were calculated taking into account the second and third position of each codon. (TIF) [file pone.0022855.s024.tif]

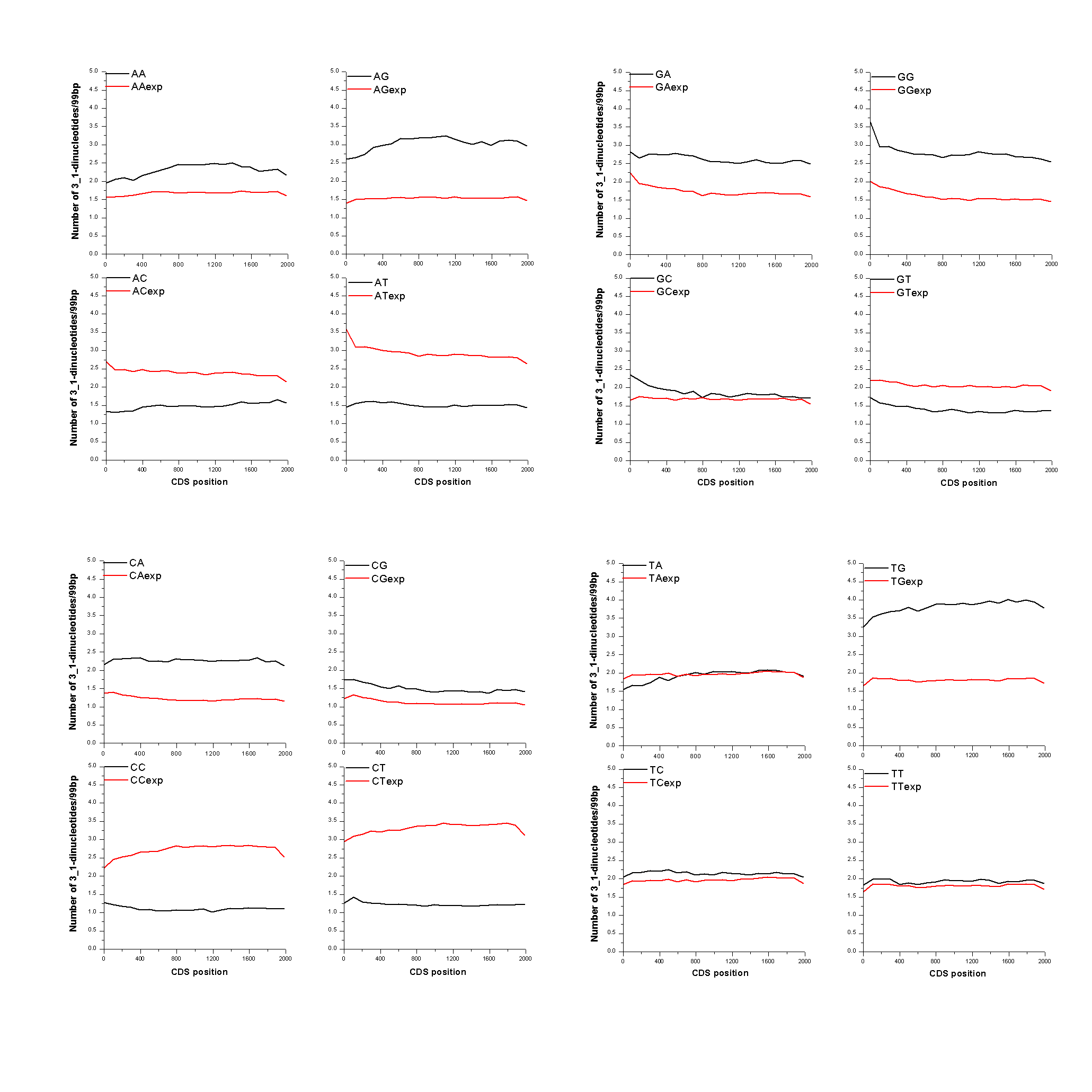

Supplement: Figure S25 — Dinucleotide content of the first 2 kb of Physcomitrella cds. The dinucleotide content were calculated taking into account the third position of a codon and the first of the subsequent codon. (TIF) [file pone.0022855.s025.tif]

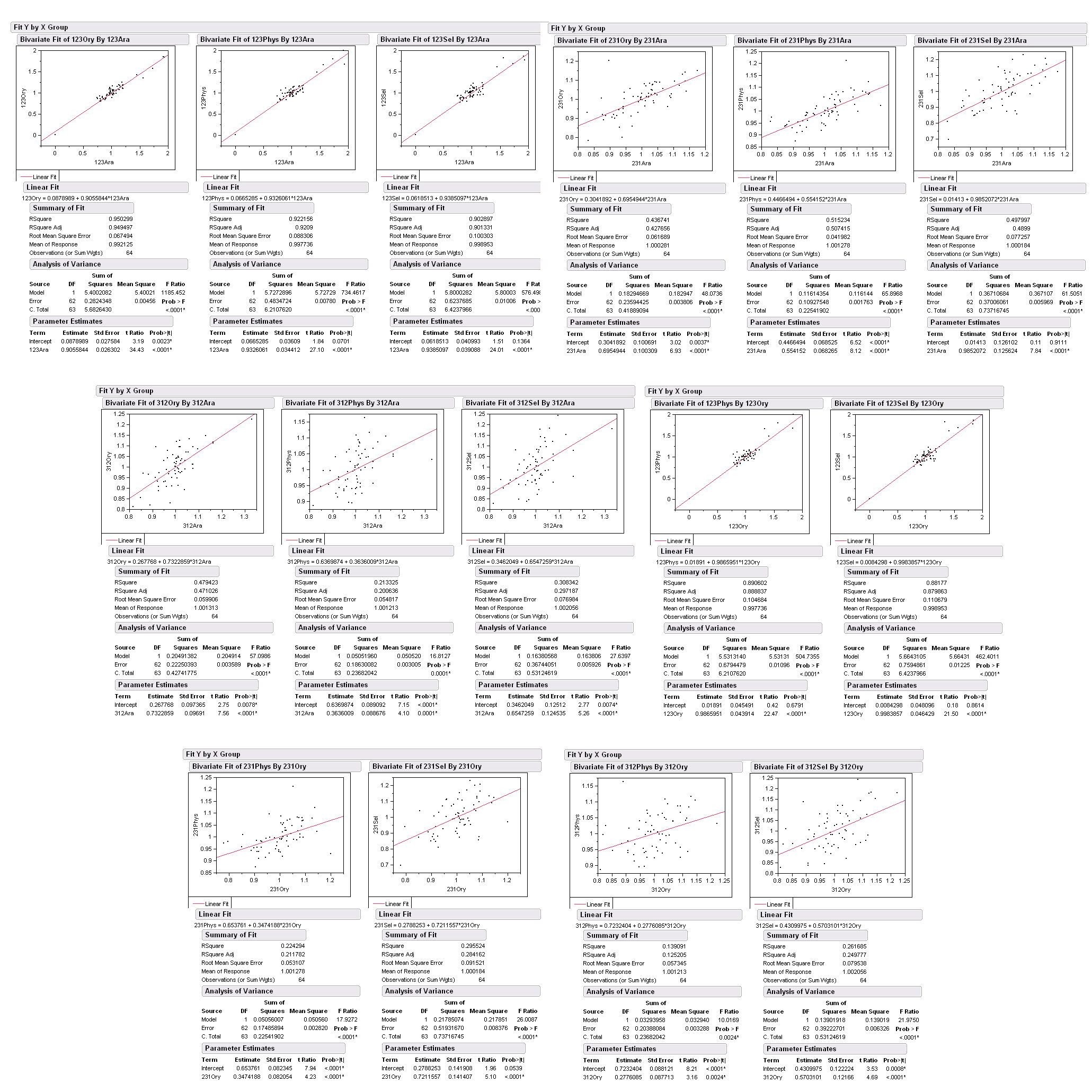

Supplement: Figure S26 — Linear fitting of the trinucleotide biases of Oryza, Arabidopsis, Physcomytrella and Selaginella. (TIF) [file pone.0022855.s026.tif]

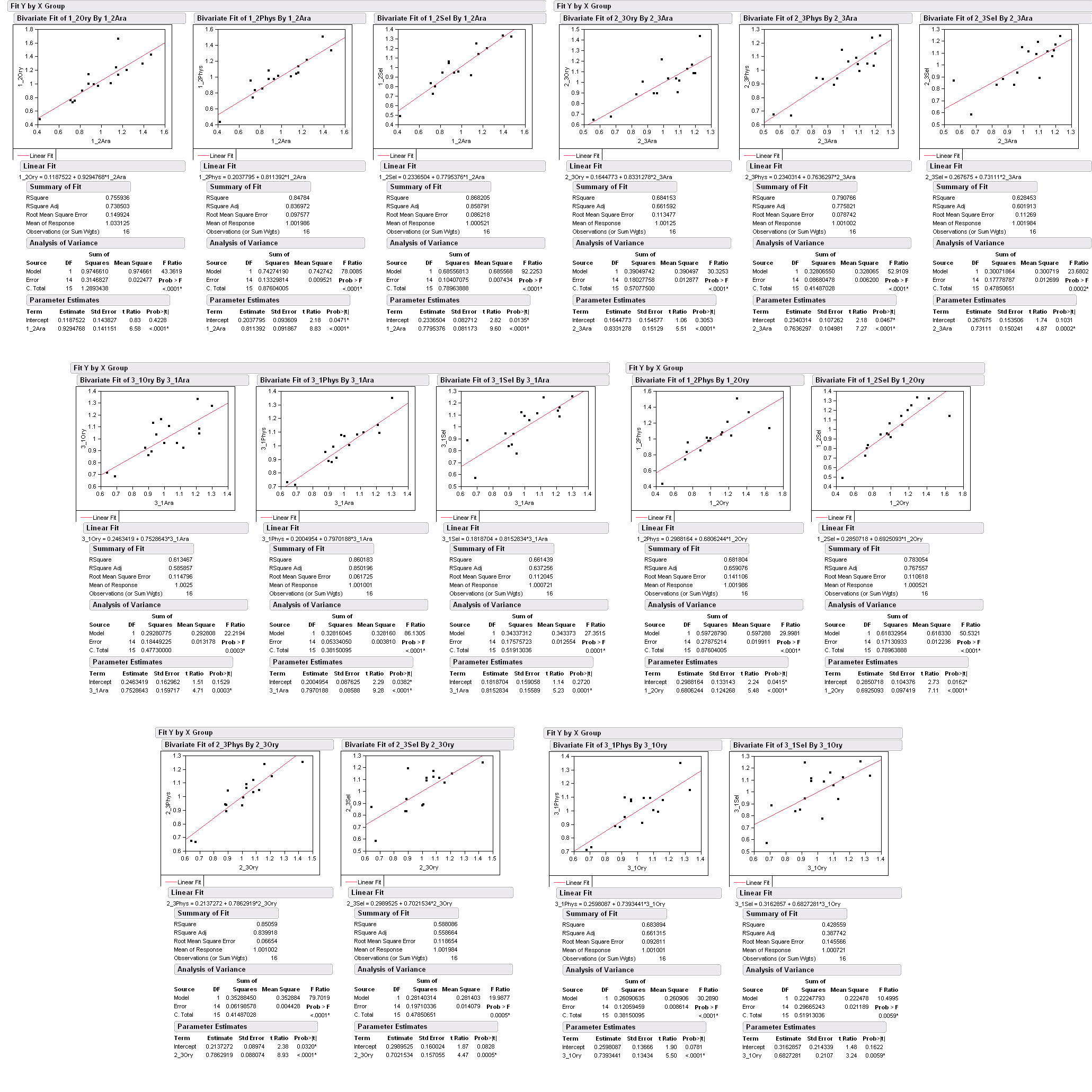

Supplement: Figure S27 — Linear fitting of the dinucleotide biases of Oryza, Arabidopsis, Physcomytrella and Selaginella. (TIF) [file pone.0022855.s027.tif]
