# Supplementary material for: Spatial Analyses of Mono, Di and Trinucleotide Trends in Plant Genes
Source: PLoS One. 2011 Aug 1;6(8):e22855. doi: 10.1371/journal.pone.0022855 (PMC3148226; doi:10.1371/journal.pone.0022855)
Supplement: Table S1 — (a) Break points and slopes of the ensemble graphs for Oryza sativa. Slopes are expressed in bp/Kbp. (b) Percentage of significant fitting to the segmented and linear regression for Ozyza sativa. (DOC) [file pone.0022855.s028.doc]

(a)

|  | Break Point | Slope 1 | Slope 2 |
| --- | --- | --- | --- |
| **Rice** |  |  |  |
| **A** | 30.6 | 0.35 | -0.67 |
| **G** | 15.8 | -1.73 | 0.00 |
| **C** | 30.6 | -0.48 | 0.66 |
| **T** | 17.5 | 1.46 | 0.00 |

(b)

|  |  |  |  |  |  | **Slope 1** |  |  |  |  |  |  |
| --- | --- | --- | --- | --- | --- | --- | --- | --- | --- | --- | --- | --- |
|  |  | **Positive** | | | |  | **Negative** | | | |  |  |
|  |  | **A** | **G** | **C** | **T** |  | **A** | **G** | **C** | **T** |  |  |
|  | | 14.1 | 1.5 | 1.1 | 10.5 |  | 7.2 | 32.6 | 43.5 | 9.5 | **Positive** |  |
| **Segmented Regression** | |  |  |  |  |  |  |  |  |  |  | **Slope 2** |
|  | | 42.2 | 14.1 | 12.5 | 40.6 |  | 0.7 | 13.7 | 13.6 | 1.3 | **Negative** |  |
| **Linear Regression** | | 13.3 | 2.3 | 1.7 | 2.2 |  | 1.6 | 11.0 | 7.8 | 12.0 |  |  |
| **Not Fitted** | | 20.9 | 24.8 | 19.7 | 23.9 |  |  |  |  |  |  |  |
