# Supplementary material for: Spatial Analyses of Mono, Di and Trinucleotide Trends in Plant Genes
Source: PLoS One. 2011 Aug 1;6(8):e22855. doi: 10.1371/journal.pone.0022855 (PMC3148226; doi:10.1371/journal.pone.0022855)
Supplement: Table S2 — (a) Average trinucleotide bias of Arabidopsis coding sequences. The trinucleotide contents were calculated for each window position and averaged over all sequences longer than 2 kb using a window of 99 bp. The γ index were calculated according to Karlin [2]. (b) Average trinucleotide bias of Oryza coding sequences. The trinucleotide contents were calculated for each windoe position and averaged over all sequences longer than 2 kb using a window of 99 bp. The γ index were calculated according to Karlin [2]. (DOC) [file pone.0022855.s029.doc]

(a)

|  | **1_2_3** | | |  | **2_3_1** | | |  | **3_1_2** | | |  |  | **1_2_3** | | |  | **2_3_1** | | |  | **3_1_2** | | |
| --- | --- | --- | --- | --- | --- | --- | --- | --- | --- | --- | --- | --- | --- | --- | --- | --- | --- | --- | --- | --- | --- | --- | --- | --- |
|  | average |  | σ |  | average |  | σ |  | average |  | σ |  |  | average |  | σ |  | average |  | σ |  | average |  | σ |
| **AAA** | 0.977 | ± | 0.017 |  | 0.876 | ± | 0.022 |  | 0.928 | ± | 0.033 |  | **CAA** | 1.130 | ± | 0.024 |  | 1.118 | ± | 0.028 |  | 1.054 | ± | 0.023 |
| **AAG** | 1.027 | ± | 0.041 |  | 1.129 | ± | 0.010 |  | 1.075 | ± | 0.019 |  | **CAG** | 1.197 | ± | 0.029 |  | 0.880 | ± | 0.028 |  | 0.849 | ± | 0.029 |
| **AAC** | 0.973 | ± | 0.026 |  | 1.001 | ± | 0.028 |  | 1.041 | ± | 0.025 |  | **CAC** | 0.858 | ± | 0.032 |  | 1.025 | ± | 0.039 |  | 1.046 | ± | 0.020 |
| **AAT** | 0.968 | ± | 0.034 |  | 0.941 | ± | 0.043 |  | 1.019 | ± | 0.045 |  | **CAT** | 0.796 | ± | 0.020 |  | 1.022 | ± | 0.022 |  | 1.009 | ± | 0.027 |
| **AGA** | 1.101 | ± | 0.033 |  | 1.034 | ± | 0.020 |  | 1.027 | ± | 0.020 |  | **CGA** | 0.891 | ± | 0.044 |  | 0.938 | ± | 0.025 |  | 1.010 | ± | 0.044 |
| **AGG** | 0.787 | ± | 0.047 |  | 0.874 | ± | 0.043 |  | 0.882 | ± | 0.022 |  | **CGG** | 1.010 | ± | 0.080 |  | 1.094 | ± | 0.057 |  | 1.162 | ± | 0.054 |
| **AGC** | 1.042 | ± | 0.046 |  | 1.082 | ± | 0.049 |  | 1.007 | ± | 0.023 |  | **CGC** | 1.013 | ± | 0.066 |  | 0.897 | ± | 0.046 |  | 0.914 | ± | 0.038 |
| **AGT** | 1.043 | ± | 0.028 |  | 1.038 | ± | 0.026 |  | 1.019 | ± | 0.031 |  | **CGT** | 1.081 | ± | 0.030 |  | 1.091 | ± | 0.049 |  | 0.912 | ± | 0.048 |
| **ACA** | 0.959 | ± | 0.024 |  | 0.999 | ± | 0.015 |  | 0.989 | ± | 0.034 |  | **CCA** | 1.014 | ± | 0.028 |  | 1.104 | ± | 0.025 |  | 1.073 | ± | 0.033 |
| **ACG** | 0.886 | ± | 0.037 |  | 0.970 | ± | 0.034 |  | 0.976 | ± | 0.053 |  | **CCG** | 1.200 | ± | 0.077 |  | 1.175 | ± | 0.068 |  | 1.330 | ± | 0.061 |
| **ACC** | 1.151 | ± | 0.055 |  | 1.041 | ± | 0.044 |  | 1.015 | ± | 0.034 |  | **CCC** | 0.805 | ± | 0.079 |  | 0.831 | ± | 0.083 |  | 0.927 | ± | 0.061 |
| **ACT** | 1.078 | ± | 0.024 |  | 1.013 | ± | 0.039 |  | 1.026 | ± | 0.043 |  | **CCT** | 0.964 | ± | 0.014 |  | 0.829 | ± | 0.038 |  | 0.859 | ± | 0.032 |
| **ATA** | 0.968 | ± | 0.066 |  | 1.022 | ± | 0.029 |  | 0.984 | ± | 0.066 |  | **CTA** | 0.906 | ± | 0.040 |  | 1.000 | ± | 0.022 |  | 1.003 | ± | 0.029 |
| **ATG** | 1.037 | ± | 0.060 |  | 1.011 | ± | 0.021 |  | 1.075 | ± | 0.053 |  | **CTG** | 0.740 | ± | 0.061 |  | 0.942 | ± | 0.016 |  | 0.960 | ± | 0.054 |
| **ATC** | 0.926 | ± | 0.048 |  | 0.942 | ± | 0.019 |  | 0.999 | ± | 0.021 |  | **CTC** | 1.207 | ± | 0.042 |  | 1.056 | ± | 0.019 |  | 1.014 | ± | 0.022 |
| **ATT** | 1.028 | ± | 0.054 |  | 1.030 | ± | 0.023 |  | 1.002 | ± | 0.030 |  | **CTT** | 1.142 | ± | 0.026 |  | 1.037 | ± | 0.020 |  | 1.000 | ± | 0.019 |
| **GAA** | 0.979 | ± | 0.021 |  | 1.042 | ± | 0.023 |  | 0.996 | ± | 0.020 |  | **TAA** | 0.000 | ± | 0.000 |  | 0.990 | ± | 0.025 |  | 0.983 | ± | 0.034 |
| **GAG** | 1.080 | ± | 0.018 |  | 1.035 | ± | 0.022 |  | 1.129 | ± | 0.026 |  | **TAG** | 0.000 | ± | 0.000 |  | 0.906 | ± | 0.021 |  | 0.970 | ± | 0.023 |
| **GAC** | 0.945 | ± | 0.017 |  | 0.930 | ± | 0.048 |  | 0.950 | ± | 0.031 |  | **TAC** | 1.914 | ± | 0.085 |  | 1.066 | ± | 0.039 |  | 1.010 | ± | 0.030 |
| **GAT** | 0.989 | ± | 0.017 |  | 0.929 | ± | 0.027 |  | 0.921 | ± | 0.024 |  | **TAT** | 1.730 | ± | 0.065 |  | 1.137 | ± | 0.048 |  | 1.059 | ± | 0.043 |
| **GGA** | 1.184 | ± | 0.021 |  | 1.009 | ± | 0.029 |  | 1.004 | ± | 0.025 |  | **TGA** | 0.000 | ± | 0.000 |  | 0.974 | ± | 0.029 |  | 0.995 | ± | 0.009 |
| **GGG** | 0.792 | ± | 0.043 |  | 0.922 | ± | 0.068 |  | 0.817 | ± | 0.057 |  | **TGG** | 1.938 | ± | 0.151 |  | 1.140 | ± | 0.042 |  | 1.095 | ± | 0.032 |
| **GGC** | 0.974 | ± | 0.073 |  | 1.049 | ± | 0.053 |  | 1.163 | ± | 0.050 |  | **TGC** | 1.007 | ± | 0.060 |  | 0.911 | ± | 0.047 |  | 0.927 | ± | 0.029 |
| **GGT** | 0.978 | ± | 0.023 |  | 1.015 | ± | 0.042 |  | 1.001 | ± | 0.034 |  | **TGT** | 0.971 | ± | 0.045 |  | 0.945 | ± | 0.025 |  | 1.023 | ± | 0.018 |
| **GCA** | 0.853 | ± | 0.028 |  | 0.915 | ± | 0.036 |  | 0.955 | ± | 0.041 |  | **TCA** | 1.451 | ± | 0.034 |  | 0.996 | ± | 0.017 |  | 1.014 | ± | 0.023 |
| **GCG** | 0.973 | ± | 0.058 |  | 0.964 | ± | 0.046 |  | 0.966 | ± | 0.053 |  | **TCG** | 1.024 | ± | 0.048 |  | 0.945 | ± | 0.046 |  | 0.890 | ± | 0.025 |
| **GCC** | 1.196 | ± | 0.026 |  | 1.136 | ± | 0.078 |  | 0.998 | ± | 0.044 |  | **TCC** | 0.810 | ± | 0.035 |  | 0.988 | ± | 0.029 |  | 1.021 | ± | 0.025 |
| **GCT** | 1.013 | ± | 0.023 |  | 1.010 | ± | 0.042 |  | 1.040 | ± | 0.025 |  | **TCT** | 0.908 | ± | 0.015 |  | 1.089 | ± | 0.034 |  | 1.014 | ± | 0.018 |
| **GTA** | 0.842 | ± | 0.025 |  | 0.988 | ± | 0.033 |  | 0.965 | ± | 0.053 |  | **TTA** | 1.564 | ± | 0.093 |  | 0.997 | ± | 0.028 |  | 1.039 | ± | 0.035 |
| **GTG** | 0.992 | ± | 0.041 |  | 1.033 | ± | 0.018 |  | 1.017 | ± | 0.045 |  | **TTG** | 1.159 | ± | 0.070 |  | 1.030 | ± | 0.013 |  | 0.961 | ± | 0.030 |
| **GTC** | 1.025 | ± | 0.030 |  | 0.967 | ± | 0.029 |  | 0.971 | ± | 0.028 |  | **TTC** | 0.835 | ± | 0.033 |  | 1.022 | ± | 0.027 |  | 1.006 | ± | 0.014 |
| **GTT** | 1.116 | ± | 0.036 |  | 1.046 | ± | 0.034 |  | 1.074 | ± | 0.025 |  | **TTT** | 0.757 | ± | 0.021 |  | 0.887 | ± | 0.019 |  | 0.950 | ± | 0.022 |

(b)

|  | **1_2_3** | | |  | **2_3_1** | | |  | **3_1_2** | | |  |  | **1_2_3** | | |  | **2_3_1** | | |  | **3_1_2** | | |
| --- | --- | --- | --- | --- | --- | --- | --- | --- | --- | --- | --- | --- | --- | --- | --- | --- | --- | --- | --- | --- | --- | --- | --- | --- |
|  | average |  | σ |  | average |  | σ |  | average |  | σ |  |  | average |  | σ |  | average |  | σ |  | average |  | σ |
| **AAA** | 0.958 | ± | 0.037 |  | 0.917 | ± | 0.020 |  | 0.971 | ± | 0.016 |  | **CAA** | 1.181 | ± | 0.060 |  | 1.069 | ± | 0.044 |  | 1.067 | ± | 0.023 |
| **AAG** | 1.059 | ± | 0.031 |  | 1.128 | ± | 0.018 |  | 1.092 | ± | 0.049 |  | **CAG** | 1.095 | ± | 0.021 |  | 0.888 | ± | 0.033 |  | 0.853 | ± | 0.023 |
| **AAC** | 0.979 | ± | 0.029 |  | 0.995 | ± | 0.031 |  | 0.988 | ± | 0.034 |  | **CAC** | 0.851 | ± | 0.050 |  | 1.080 | ± | 0.035 |  | 1.067 | ± | 0.020 |
| **AAT** | 0.954 | ± | 0.031 |  | 0.925 | ± | 0.028 |  | 0.971 | ± | 0.023 |  | **CAT** | 0.847 | ± | 0.022 |  | 1.009 | ± | 0.023 |  | 0.993 | ± | 0.020 |
| **AGA** | 1.133 | ± | 0.031 |  | 1.044 | ± | 0.028 |  | 1.036 | ± | 0.017 |  | **CGA** | 0.845 | ± | 0.073 |  | 0.796 | ± | 0.069 |  | 0.910 | ± | 0.023 |
| **AGG** | 0.907 | ± | 0.044 |  | 1.002 | ± | 0.018 |  | 0.962 | ± | 0.060 |  | **CGG** | 0.981 | ± | 0.055 |  | 1.025 | ± | 0.024 |  | 1.111 | ± | 0.041 |
| **AGC** | 0.968 | ± | 0.042 |  | 0.959 | ± | 0.023 |  | 0.946 | ± | 0.035 |  | **CGC** | 1.151 | ± | 0.050 |  | 1.201 | ± | 0.059 |  | 1.092 | ± | 0.035 |
| **AGT** | 1.054 | ± | 0.036 |  | 0.979 | ± | 0.028 |  | 1.012 | ± | 0.026 |  | **CGT** | 0.969 | ± | 0.071 |  | 1.005 | ± | 0.045 |  | 0.948 | ± | 0.021 |
| **ACA** | 0.987 | ± | 0.042 |  | 1.007 | ± | 0.017 |  | 1.054 | ± | 0.050 |  | **CCA** | 1.048 | ± | 0.057 |  | 1.092 | ± | 0.018 |  | 1.011 | ± | 0.025 |
| **ACG** | 0.819 | ± | 0.055 |  | 0.855 | ± | 0.029 |  | 0.828 | ± | 0.065 |  | **CCG** | 1.082 | ± | 0.042 |  | 1.146 | ± | 0.027 |  | 1.222 | ± | 0.042 |
| **ACC** | 1.161 | ± | 0.061 |  | 1.088 | ± | 0.035 |  | 1.093 | ± | 0.053 |  | **CCC** | 0.784 | ± | 0.039 |  | 0.780 | ± | 0.038 |  | 0.906 | ± | 0.045 |
| **ACT** | 1.048 | ± | 0.041 |  | 1.067 | ± | 0.032 |  | 0.975 | ± | 0.034 |  | **CCT** | 1.080 | ± | 0.065 |  | 0.927 | ± | 0.041 |  | 0.968 | ± | 0.042 |
| **ATA** | 1.113 | ± | 0.070 |  | 1.029 | ± | 0.039 |  | 0.947 | ± | 0.044 |  | **CTA** | 0.858 | ± | 0.043 |  | 1.029 | ± | 0.037 |  | 1.118 | ± | 0.031 |
| **ATG** | 0.973 | ± | 0.058 |  | 0.989 | ± | 0.023 |  | 1.009 | ± | 0.040 |  | **CTG** | 0.877 | ± | 0.029 |  | 0.946 | ± | 0.028 |  | 0.986 | ± | 0.058 |
| **ATC** | 0.945 | ± | 0.051 |  | 0.958 | ± | 0.018 |  | 1.000 | ± | 0.018 |  | **CTC** | 1.175 | ± | 0.033 |  | 1.087 | ± | 0.041 |  | 0.974 | ± | 0.025 |
| **ATT** | 1.020 | ± | 0.057 |  | 1.048 | ± | 0.028 |  | 1.038 | ± | 0.043 |  | **CTT** | 1.064 | ± | 0.034 |  | 0.988 | ± | 0.025 |  | 0.965 | ± | 0.017 |
| **GAA** | 1.016 | ± | 0.033 |  | 1.027 | ± | 0.056 |  | 0.999 | ± | 0.018 |  | **TAA** | 0.000 | ± | 0.001 |  | 0.983 | ± | 0.023 |  | 0.907 | ± | 0.030 |
| **GAG** | 1.037 | ± | 0.029 |  | 1.075 | ± | 0.054 |  | 1.145 | ± | 0.040 |  | **TAG** | 0.000 | ± | 0.000 |  | 0.904 | ± | 0.026 |  | 0.965 | ± | 0.035 |
| **GAC** | 0.929 | ± | 0.035 |  | 0.850 | ± | 0.048 |  | 0.927 | ± | 0.033 |  | **TAC** | 1.837 | ± | 0.031 |  | 1.053 | ± | 0.045 |  | 1.047 | ± | 0.032 |
| **GAT** | 0.999 | ± | 0.019 |  | 0.943 | ± | 0.033 |  | 0.930 | ± | 0.017 |  | **TAT** | 1.567 | ± | 0.058 |  | 1.138 | ± | 0.035 |  | 1.130 | ± | 0.021 |
| **GGA** | 1.240 | ± | 0.039 |  | 1.079 | ± | 0.069 |  | 1.022 | ± | 0.024 |  | **TGA** | 0.000 | ± | 0.000 |  | 1.010 | ± | 0.012 |  | 1.018 | ± | 0.017 |
| **GGG** | 0.790 | ± | 0.018 |  | 0.910 | ± | 0.034 |  | 0.809 | ± | 0.022 |  | **TGG** | 1.827 | ± | 0.059 |  | 1.040 | ± | 0.025 |  | 1.083 | ± | 0.035 |
| **GGC** | 1.042 | ± | 0.016 |  | 1.020 | ± | 0.058 |  | 1.115 | ± | 0.029 |  | **TGC** | 0.881 | ± | 0.051 |  | 0.916 | ± | 0.021 |  | 0.885 | ± | 0.035 |
| **GGT** | 1.013 | ± | 0.033 |  | 1.010 | ± | 0.041 |  | 1.013 | ± | 0.025 |  | **TGT** | 0.933 | ± | 0.034 |  | 1.034 | ± | 0.025 |  | 1.028 | ± | 0.015 |
| **GCA** | 0.833 | ± | 0.032 |  | 0.898 | ± | 0.022 |  | 0.931 | ± | 0.019 |  | **TCA** | 1.413 | ± | 0.050 |  | 1.005 | ± | 0.011 |  | 1.042 | ± | 0.029 |
| **GCG** | 1.094 | ± | 0.034 |  | 1.054 | ± | 0.027 |  | 1.075 | ± | 0.038 |  | **TCG** | 1.045 | ± | 0.039 |  | 1.000 | ± | 0.045 |  | 0.818 | ± | 0.057 |
| **GCC** | 1.162 | ± | 0.034 |  | 1.091 | ± | 0.035 |  | 0.979 | ± | 0.036 |  | **TCC** | 0.814 | ± | 0.035 |  | 1.011 | ± | 0.036 |  | 1.092 | ± | 0.065 |
| **GCT** | 0.956 | ± | 0.043 |  | 0.961 | ± | 0.029 |  | 1.028 | ± | 0.020 |  | **TCT** | 0.921 | ± | 0.017 |  | 1.014 | ± | 0.023 |  | 0.987 | ± | 0.028 |
| **GTA** | 0.881 | ± | 0.040 |  | 0.929 | ± | 0.027 |  | 1.041 | ± | 0.045 |  | **TTA** | 1.335 | ± | 0.049 |  | 0.996 | ± | 0.028 |  | 0.890 | ± | 0.032 |
| **GTG** | 1.077 | ± | 0.046 |  | 1.071 | ± | 0.017 |  | 1.123 | ± | 0.040 |  | **TTG** | 1.124 | ± | 0.084 |  | 1.021 | ± | 0.018 |  | 0.906 | ± | 0.028 |
| **GTC** | 0.934 | ± | 0.038 |  | 0.908 | ± | 0.036 |  | 0.976 | ± | 0.020 |  | **TTC** | 0.904 | ± | 0.034 |  | 1.012 | ± | 0.018 |  | 1.041 | ± | 0.022 |
| **GTT** | 1.076 | ± | 0.036 |  | 1.086 | ± | 0.017 |  | 0.991 | ± | 0.025 |  | **TTT** | 0.850 | ± | 0.060 |  | 0.911 | ± | 0.030 |  | 1.028 | ± | 0.028 |
