# Supplementary material for: Spatial Analyses of Mono, Di and Trinucleotide Trends in Plant Genes
Source: PLoS One. 2011 Aug 1;6(8):e22855. doi: 10.1371/journal.pone.0022855 (PMC3148226; doi:10.1371/journal.pone.0022855)
Supplement: Table S3 — Average trinucleotide bias of intron sequences. The trinucleotide contents were calculated for each window position and averaged over all sequences. (DOC) [file pone.0022855.s030.doc]

| *Arabidopsis thaliana* | | | | | | | |  |  | *Oryza sativa* | | | | | | |  |
| --- | --- | --- | --- | --- | --- | --- | --- | --- | --- | --- | --- | --- | --- | --- | --- | --- | --- |
|  | **γindex** | | |  |  | **γindex** | | |  | **γindex** | | |  |  | **γindex** | | |
|  | average |  | σ |  |  | average |  | σ |  | average |  | σ |  |  | average |  | σ |
| **AAA** | 0.986 | ± | 0.014 |  | **CAA** | 1.011 | ± | 0.026 | **AAA** | 1.028 | ± | 0.029 |  | **CAA** | 0.961 | ± | 0.022 |
| **AAG** | 0.992 | ± | 0.015 |  | **CAG** | 0.878 | ± | 0.025 | **AAG** | 0.930 | ± | 0.026 |  | **CAG** | 0.948 | ± | 0.014 |
| **AAC** | 1.012 | ± | 0.029 |  | **CAC** | 1.012 | ± | 0.023 | **AAC** | 1.025 | ± | 0.027 |  | **CAC** | 1.034 | ± | 0.029 |
| **AAT** | 0.991 | ± | 0.019 |  | **CAT** | 1.040 | ± | 0.024 | **AAT** | 0.981 | ± | 0.015 |  | **CAT** | 1.057 | ± | 0.015 |
| **AGA** | 0.958 | ± | 0.023 |  | **CGA** | 1.028 | ± | 0.055 | **AGA** | 0.955 | ± | 0.018 |  | **CGA** | 0.985 | ± | 0.029 |
| **AGG** | 0.923 | ± | 0.058 |  | **CGG** | 1.072 | ± | 0.140 | **AGG** | 0.986 | ± | 0.020 |  | **CGG** | 1.049 | ± | 0.059 |
| **AGC** | 1.052 | ± | 0.067 |  | **CGC** | 0.812 | ± | 0.050 | **AGC** | 0.993 | ± | 0.017 |  | **CGC** | 0.995 | ± | 0.022 |
| **AGT** | 1.042 | ± | 0.043 |  | **CGT** | 1.016 | ± | 0.023 | **AGT** | 1.053 | ± | 0.021 |  | **CGT** | 0.983 | ± | 0.034 |
| **ACA** | 0.958 | ± | 0.045 |  | **CCA** | 1.115 | ± | 0.024 | **ACA** | 0.987 | ± | 0.074 |  | **CCA** | 1.027 | ± | 0.019 |
| **ACG** | 1.054 | ± | 0.038 |  | **CCG** | 1.151 | ± | 0.085 | **ACG** | 0.962 | ± | 0.044 |  | **CCG** | 1.070 | ± | 0.040 |
| **ACC** | 1.045 | ± | 0.048 |  | **CCC** | 0.968 | ± | 0.044 | **ACC** | 1.004 | ± | 0.021 |  | **CCC** | 0.960 | ± | 0.023 |
| **ACT** | 1.007 | ± | 0.038 |  | **CCT** | 0.887 | ± | 0.026 | **ACT** | 1.018 | ± | 0.031 |  | **CCT** | 0.975 | ± | 0.014 |
| **ATA** | 1.019 | ± | 0.036 |  | **CTA** | 0.963 | ± | 0.036 | **ATA** | 0.990 | ± | 0.033 |  | **CTA** | 1.015 | ± | 0.022 |
| **ATG** | 1.032 | ± | 0.026 |  | **CTG** | 1.017 | ± | 0.035 | **ATG** | 1.062 | ± | 0.012 |  | **CTG** | 1.025 | ± | 0.020 |
| **ATC** | 0.971 | ± | 0.025 |  | **CTC** | 1.024 | ± | 0.016 | **ATC** | 1.005 | ± | 0.031 |  | **CTC** | 1.033 | ± | 0.015 |
| **ATT** | 1.000 | ± | 0.026 |  | **CTT** | 1.007 | ± | 0.014 | **ATT** | 0.983 | ± | 0.010 |  | **CTT** | 0.957 | ± | 0.009 |
| **GAA** | 1.111 | ± | 0.026 |  | **TAA** | 0.949 | ± | 0.015 | **GAA** | 1.051 | ± | 0.038 |  | **TAA** | 0.975 | ± | 0.030 |
| **GAG** | 1.087 | ± | 0.056 |  | **TAG** | 1.103 | ± | 0.050 | **GAG** | 1.097 | ± | 0.072 |  | **TAG** | 1.125 | ± | 0.058 |
| **GAC** | 1.044 | ± | 0.022 |  | **TAC** | 1.013 | ± | 0.051 | **GAC** | 1.016 | ± | 0.028 |  | **TAC** | 1.011 | ± | 0.044 |
| **GAT** | 1.070 | ± | 0.030 |  | **TAT** | 0.997 | ± | 0.037 | **GAT** | 1.115 | ± | 0.055 |  | **TAT** | 0.960 | ± | 0.066 |
| **GGA** | 0.984 | ± | 0.054 |  | **TGA** | 1.041 | ± | 0.015 | **GGA** | 1.031 | ± | 0.025 |  | **TGA** | 1.042 | ± | 0.017 |
| **GGG** | 1.049 | ± | 0.144 |  | **TGG** | 1.006 | ± | 0.043 | **GGG** | 1.021 | ± | 0.028 |  | **TGG** | 0.982 | ± | 0.014 |
| **GGC** | 1.081 | ± | 0.046 |  | **TGC** | 0.988 | ± | 0.023 | **GGC** | 1.039 | ± | 0.057 |  | **TGC** | 0.994 | ± | 0.021 |
| **GGT** | 0.971 | ± | 0.027 |  | **TGT** | 0.983 | ± | 0.025 | **GGT** | 0.943 | ± | 0.017 |  | **TGT** | 0.987 | ± | 0.012 |
| **GCA** | 0.966 | ± | 0.043 |  | **TCA** | 1.021 | ± | 0.010 | **GCA** | 0.962 | ± | 0.017 |  | **TCA** | 1.038 | ± | 0.034 |
| **GCG** | 0.876 | ± | 0.057 |  | **TCG** | 0.955 | ± | 0.023 | **GCG** | 1.014 | ± | 0.036 |  | **TCG** | 0.962 | ± | 0.030 |
| **GCC** | 1.074 | ± | 0.082 |  | **TCC** | 0.966 | ± | 0.024 | **GCC** | 1.086 | ± | 0.060 |  | **TCC** | 0.981 | ± | 0.029 |
| **GCT** | 1.011 | ± | 0.018 |  | **TCT** | 1.024 | ± | 0.009 | **GCT** | 0.986 | ± | 0.031 |  | **TCT** | 1.009 | ± | 0.032 |
| **GTA** | 0.964 | ± | 0.037 |  | **TTA** | 1.026 | ± | 0.018 | **GTA** | 0.996 | ± | 0.034 |  | **TTA** | 1.010 | ± | 0.014 |
| **GTG** | 1.003 | ± | 0.029 |  | **TTG** | 0.973 | ± | 0.015 | **GTG** | 1.009 | ± | 0.013 |  | **TTG** | 0.946 | ± | 0.015 |
| **GTC** | 0.993 | ± | 0.015 |  | **TTC** | 1.007 | ± | 0.017 | **GTC** | 0.950 | ± | 0.043 |  | **TTC** | 0.988 | ± | 0.018 |
| **GTT** | 1.031 | ± | 0.012 |  | **TTT** | 0.982 | ± | 0.010 | **GTT** | 1.028 | ± | 0.016 |  | **TTT** | 1.019 | ± | 0.008 |
