# Supplementary material for: Spatial Analyses of Mono, Di and Trinucleotide Trends in Plant Genes
Source: PLoS One. 2011 Aug 1;6(8):e22855. doi: 10.1371/journal.pone.0022855 (PMC3148226; doi:10.1371/journal.pone.0022855)
Supplement: Table S4 — Average trinucleotide bias of Physcomytrella and Selaginella coding sequences. The trinucleotide contents were calculated for each windoe position and averaged over all sequences longer than 2 kb using a window of 99 bp. The γ index were calculated according to Karlin [2]. (DOC) [file pone.0022855.s031.doc]

|  | ***Physcomytrella*** | | | |  | ***Selaginella*** | | | |
| --- | --- | --- | --- | --- | --- | --- | --- | --- | --- |
| **Nucleotide** | **All** | **123** | **231** | **312** |  | **All** | **123** | **231** | **312** |
| AAA | 0.95 | **1.00** | 0.94 | 0.94 |  | 0.97 | **1.01** | 0.94 | **1.01** |
| AAG | **1.12** | **1.12** | **1.08** | **1.06** |  | **1.13** | **1.10** | **1.13** | **1.06** |
| AAC | 0.95 | 0.90 | 0.94 | **1.01** |  | 0.92 | 0.89 | 0.91 | 0.98 |
| AAT | 0.95 | 0.93 | 0.98 | **1.03** |  | 0.89 | 0.89 | 0.88 | 0.96 |
| AGA | **1.04** | **1.01** | **1.01** | **1.05** |  | 0.97 | 0.94 | 0.99 | 0.96 |
| AGG | 0.91 | 0.84 | 0.93 | 0.95 |  | 0.88 | 0.86 | 0.89 | 0.92 |
| AGC | **1.06** | **1.14** | **1.09** | **1.01** |  | **1.09** | **1.12** | **1.11** | **1.02** |
| AGT | 0.99 | **1.05** | **1.00** | 0.94 |  | **1.08** | **1.08** | **1.07** | **1.09** |
| ACA | **1.02** | **1.05** | 0.99 | 0.99 |  | **1.04** | 0.99 | **1.00** | **1.04** |
| ACG | 0.99 | 0.98 | **1.01** | **1.02** |  | **1.02** | 0.98 | **1.04** | **1.01** |
| ACC | **1.06** | **1.03** | **1.07** | **1.09** |  | 0.88 | 0.94 | 0.87 | 0.94 |
| ACT | 0.96 | 0.97 | 0.98 | 0.92 |  | **1.07** | **1.13** | **1.07** | **1.04** |
| ATA | **1.01** | 0.95 | **1.07** | 0.89 |  | **1.04** | **1.09** | **1.05** | 0.92 |
| ATG | 0.95 | 0.92 | 0.98 | 0.96 |  | 0.95 | 0.93 | 0.99 | **1.03** |
| ATC | 0.95 | 0.99 | 0.93 | 0.92 |  | **1.02** | **1.02** | **1.00** | **1.02** |
| ATT | **1.08** | **1.11** | **1.05** | **1.11** |  | 0.99 | **1.03** | 0.97 | **1.00** |
| GAA | 0.98 | 0.93 | 0.98 | **1.01** |  | 0.92 | 0.91 | 0.98 | 0.93 |
| GAG | **1.08** | **1.06** | **1.08** | **1.10** |  | **1.10** | **1.05** | **1.04** | **1.15** |
| GAC | 0.96 | 0.99 | 0.96 | 0.93 |  | 0.97 | 0.99 | 0.87 | 0.97 |
| GAT | 0.95 | **1.00** | 0.91 | 0.91 |  | **1.00** | **1.02** | **1.07** | 0.98 |
| GGA | **1.03** | **1.15** | **1.01** | 0.98 |  | **1.16** | **1.22** | **1.13** | **1.08** |
| GGG | 0.88 | 0.85 | 0.94 | 0.88 |  | 0.79 | 0.77 | 0.86 | 0.82 |
| GGC | **1.04** | 0.97 | **1.04** | **1.05** |  | **1.09** | **1.02** | **1.10** | **1.07** |
| GGT | **1.04** | **1.00** | **1.05** | **1.06** |  | 0.92 | 0.98 | 0.87 | 0.92 |
| GCA | 0.93 | 0.90 | 0.95 | 0.97 |  | 0.86 | 0.85 | 0.85 | 0.91 |
| GCG | 0.99 | **1.00** | 0.99 | 0.97 |  | 0.94 | 0.93 | 0.95 | 0.93 |
| GCC | **1.05** | **1.08** | **1.07** | **1.00** |  | **1.10** | **1.14** | **1.20** | **0.94** |
| GCT | **1.02** | **1.03** | **1.00** | **1.06** |  | **1.09** | **1.04** | **1.11** | **1.12** |
| GTA | 0.97 | 0.97 | 0.98 | 0.99 |  | 0.98 | 0.88 | **1.03** | **1.00** |
| GTG | **1.06** | **1.07** | **1.02** | **1.08** |  | **1.18** | **1.16** | **1.20** | **1.18** |
| GTC | **1.00** | 0.98 | 0.98 | **1.06** |  | 0.92 | 0.93 | 0.89 | **1.06** |
| GTT | **1.01** | **1.01** | **1.04** | **1.02** |  | 0.95 | **1.00** | 0.85 | 0.92 |
| CAA | **1.09** | **1.14** | **1.08** | **1.04** |  | **1.07** | **1.10** | **1.05** | 0.98 |
| CAG | 0.98 | **1.10** | 0.94 | 0.95 |  | 0.95 | **1.09** | 0.90 | 0.94 |
| CAC | 0.98 | 0.85 | **1.00** | **1.04** |  | 0.99 | 0.87 | **1.12** | **1.09** |
| CAT | 0.92 | 0.82 | 0.99 | 0.96 |  | 0.96 | 0.84 | **1.02** | 0.98 |
| CGA | 0.99 | **1.10** | 0.98 | **1.01** |  | 0.97 | **1.02** | 0.94 | **1.00** |
| CGG | **1.01** | 0.99 | **1.02** | **1.04** |  | **1.01** | **1.03** | **1.03** | 0.95 |
| CGC | **0.95** | 0.93 | 0.93 | 0.92 |  | 0.94 | 0.88 | 0.97 | 0.94 |
| CGT | **1.04** | 0.95 | **1.08** | **1.04** |  | **1.10** | **1.07** | **1.11** | **1.08** |
| CCA | 0.99 | 0.90 | **1.00** | **1.00** |  | **1.12** | **1.13** | **1.16** | **1.12** |
| CCG | **1.01** | **1.00** | **1.02** | **1.03** |  | **1.09** | 0.95 | **1.12** | **1.18** |
| CCC | 0.97 | **1.05** | 0.97 | 0.98 |  | 0.82 | 0.87 | 0.69 | 0.84 |
| CCT | **1.00** | **1.05** | 0.95 | **1.00** |  | 0.89 | **1.01** | 0.81 | 0.90 |
| CTA | 0.95 | 0.82 | **1.00** | 0.94 |  | 0.93 | 0.87 | 0.95 | 0.98 |
| CTG | 0.98 | 0.91 | 0.99 | **1.05** |  | 0.95 | 0.92 | 0.92 | 0.99 |
| CTC | **1.09** | **1.16** | **1.08** | **1.07** |  | **1.13** | **1.18** | **1.09** | **1.08** |
| CTT | **1.01** | **1.15** | 0.96 | 0.94 |  | **1.05** | **1.04** | **1.08** | **1.03** |
| TAA | 0.85 | 0.00 | 0.95 | 0.94 |  | 0.84 | 0.00 | 0.98 | 0.96 |
| TAG | 0.69^†^ | 0.00 | 0.87 | 0.90 |  | 0.58^†^ | 0.00 | 0.87 | 0.84 |
| TAC | **1.30** | **1.98** | **1.21** | **1.09** |  | **1.43** | **1.85** | **1.23** | **1.07** |
| TAT | **1.27** | **1.79** | **1.12** | **1.11** |  | **1.28** | **1.68** | **1.09** | **1.18** |
| TGA | 0.97 | 0.00 | **1.02** | **1.00** |  | 0.93 | 0.00 | 0.99 | 0.98 |
| TGG | **1.17** | **1.67** | **1.07** | **1.08** |  | **1.32** | **1.76** | **1.17** | **1.24** |
| TGC | 0.92 | 0.96 | 0.91 | 0.97 |  | 0.85 | 0.90 | 0.84 | 0.92 |
| TGT | 0.95 | 0.99 | 0.96 | **1.00** |  | 0.94 | 0.88 | 0.95 | 0.97 |
| TCA | **1.11** | **1.48** | **1.06** | **1.04** |  | **1.12** | **1.60** | **1.02** | **1.08** |
| TCG | **1.02** | **1.04** | 0.99 | 0.99 |  | 0.99 | **1.21** | 0.93 | 0.94 |
| TCC | 0.91 | 0.81 | 0.92 | 0.95 |  | **1.08** | 0.93 | 1.15 | **1.14** |
| TCT | 0.99 | 0.91 | **1.06** | **1.00** |  | 0.90 | 0.78 | 0.98 | 0.89 |
| TTA | **1.12** | **1.65*** | 0.94 | **1.16** |  | **1.15** | **1.81*** | **1.01** | **1.13** |
| TTG | **1.02** | **1.06** | **1.03** | 0.95 |  | 0.95 | **1.01** | 0.98 | 0.83 |
| TTC | 0.95 | 0.83 | **1.00** | 0.97 |  | 0.89 | 0.81 | 0.96 | 0.87 |
| TTT | 0.90 | 0.77 | 0.95 | 0.94 |  | **1.01** | 0.92 | **1.00** | **1.03** |
